# Flavivirus genome recoding by codon optimisation confers genetically stable *in vivo* attenuation in both mice and mosquitoes

**DOI:** 10.1371/journal.ppat.1011753

**Published:** 2023-10-26

**Authors:** Wei-Xin Chin, Hao Yuin Kong, Isabelle Xin Yu Zhu, Zi Yun Teo, Regina Faruk, Regina Ching Hua Lee, Si Xian Ho, Zhen Qin Aw, Bowen Yi, Xin Jun Hou, Antson Kiat Yee Tan, Thinesshwary Yogarajah, Roland G. Huber, Yu Cai, Yue Wan, Justin Jang Hann Chu

**Affiliations:** 1 Laboratory of Molecular RNA Virology and Antiviral Strategies, Department of Microbiology and Immunology and Infectious Diseases Translational Research Programme, Yong Loo Lin School of Medicine, National University Health System, National University of Singapore, Singapore; 2 NUSMed Biosafety Level 3 Core Facility, Yong Loo Lin School of Medicine, National University of Singapore, Singapore; 3 Temasek Life Sciences Laboratory, 1 Research Link, National University of Singapore, Singapore; 4 Department of Biological Sciences, National University of Singapore, Singapore; 5 Genome Institute of Singapore, Agency for Science, Technology and Research (A*STAR), Singapore; 6 Bioinformatics Institute, Agency for Science, Technology and Research (A*STAR), Singapore; 7 Institute of Molecular and Cell Biology, Agency for Science, Technology and Research (A*STAR), Singapore; Universite Paris Diderot, FRANCE

## Abstract

Virus genome recoding is an attenuation method that confers genetically stable attenuation by rewriting a virus genome with numerous silent mutations. Prior flavivirus genome recoding attempts utilised codon deoptimisation approaches. However, these codon deoptimisation approaches act in a species dependent manner and were unable to confer flavivirus attenuation in mosquito cells or in mosquito animal models. To overcome these limitations, we performed flavivirus genome recoding using the contrary approach of codon optimisation. The genomes of flaviviruses such as dengue virus type 2 (DENV2) and Zika virus (ZIKV) contain functional RNA elements that regulate viral replication. We hypothesised that flavivirus genome recoding by codon optimisation would introduce silent mutations that disrupt these RNA elements, leading to decreased replication efficiency and attenuation. We chose DENV2 and ZIKV as representative flaviviruses and recoded them by codon optimising their genomes for human expression. Our study confirms that this recoding approach of codon optimisation does translate into reduced replication efficiency in mammalian, human, and mosquito cells as well as *in vivo* attenuation in both mice and mosquitoes. *In silico* modelling and RNA SHAPE analysis confirmed that DENV2 recoding resulted in the extensive disruption of genomic structural elements. Serial passaging of recoded DENV2 resulted in the emergence of rescue or adaptation mutations, but no reversion mutations. These rescue mutations were unable to rescue the delayed replication kinetics and *in vivo* attenuation of recoded DENV2, demonstrating that recoding confers genetically stable attenuation. Therefore, our recoding approach is a reliable attenuation method with potential applications for developing flavivirus vaccines.

## Author summary

The mosquito-borne flaviviruses such as dengue virus (DENV) and Zika virus (ZIKV) have established themselves as major human pathogens. Live attenuated vaccines are seen as the most effective method for preventing flavivirus infection. Flavivirus genome recoding has emerged as a next-generation vaccine development method that acts by rewriting the flavivirus genome. Previous flavivirus genome recoding attempts were based on deoptimising the flavivirus genome. However, these deoptimised flaviviruses were found to be attenuated in a species dependent manner. For example, deoptimised DENV and ZIKV did not demonstrate attenuation in mosquito cells or mosquito animal models, which is undesirable because these mosquito-borne flaviviruses should be attenuated in their mosquito vector to prevent vaccine escape. To overcome these limitations, we adopted a flavivirus genome recoding approach based on the contrary approach of optimising the flavivirus genome and applied it to DENV2 and ZIKV. We found that this genome recoding approach of codon optimisation could confer attenuation in both mouse and mosquito animal models. This indicates that our flavivirus genome recoding approach may be used as a reliable method to construct attenuated vaccine backbones for the mosquito-borne-flaviviruses in general.

## Introduction

The mosquito-borne flaviviruses have emerged as major threats to human health and quality of life [1,2]. There are several established live attenuated vaccines for flaviviruses, as well as live dengue virus (DENV) vaccines that have shown promise in phase II & III clinical trials [3,4]. Live attenuated flavivirus vaccines are considered safe and effective at protecting against flavivirus infection as they can confer life-long immunity with a single dose [3,4]. However, that there is no consistent or reliable method for generating a sufficiently attenuated flavivirus vaccine strain; methods that have proven successful in producing attenuated vaccine strains for some flaviviruses have failed to produce attenuated vaccine strains for other flaviviruses [4–11]. Even rationally designed live vaccines can suffer from the same problems of inconsistency and unpredictability [9–11].

An alternative rational design approach is synonymous virus genome recoding. Virus genome recoding is an attenuation method that typically involves deoptimising a virus genome by altering the frequencies of favourable codons, unfavourable codons, or CpG and UpA dinucleotides [8,12–25]. For flaviviruses, deoptimisation for a human host is usually performed by optimising the virus for an insect or mosquito host, but this leads to inconsistent results that are highly dependent on cell type or animal species [13,14,19,20,22,26]. For example, these deoptimisation approaches do not affect DENV replication in mosquito cells or mosquitoes [13,19]. This lack of effect in mosquitos is especially undesirable because vaccines for the mosquito-borne flaviviruses must lack transmissibility by their mosquito vectors [27–30].

We propose an alternative approach to virus genome recoding. The flavivirus genome contains many functional RNA elements that are essential for efficient virus replication [26,31–40]. These functional RNA elements may take the form of pseudoknots, RNA secondary structures, or long-range RNA interactions [26,31–40]. For example, the capsid coding region contains conserved RNA elements such as the capsid coding region hairpin element (cHP), the 5′ cyclisation sequence (5′CS), and the downstream of 5′ cyclization sequence pseudoknot (DCS-PK) [32,35,40]. Some RNA elements play a role in the cyclisation of the viral RNA genome, which is required for the virus genome to transition from a linear protein translation state to a circularised RNA replication state [32,35–40]. Silent mutations that target the cHP, 5′CS, or DCS-PK elements can inhibit genome cyclisation, which in turn reduces viral RNA replication efficiency [32,35,40]. Therefore, we hypothesised that a recoding approach that targets these RNA elements would lead to reduced replication efficiency and attenuation. Furthermore, because the function of these RNA elements is something inherent to flaviviruses, the attenuation mechanism should function regardless of cell type or animal species. For this purpose, we chose codon optimisation as a method of introducing a sufficient number of well-spaced silent mutations to disrupt the sequence, structure and function of flavivirus RNA elements.

Therefore, aim of this study is to demonstrate a flavivirus genome recoding approach that can produce attenuated dengue virus type 2 (DENV2) and Zika virus (ZIKV) strains. This virus genome recoding approach is to codon optimise the flavivirus protein coding region, leading to the disruption of functional RNA elements that are essential for efficient virus replication [26,31–40].

## Results

### Recoded DENV2-EGFP clones have reduced replication efficiency

**Fig 1:**
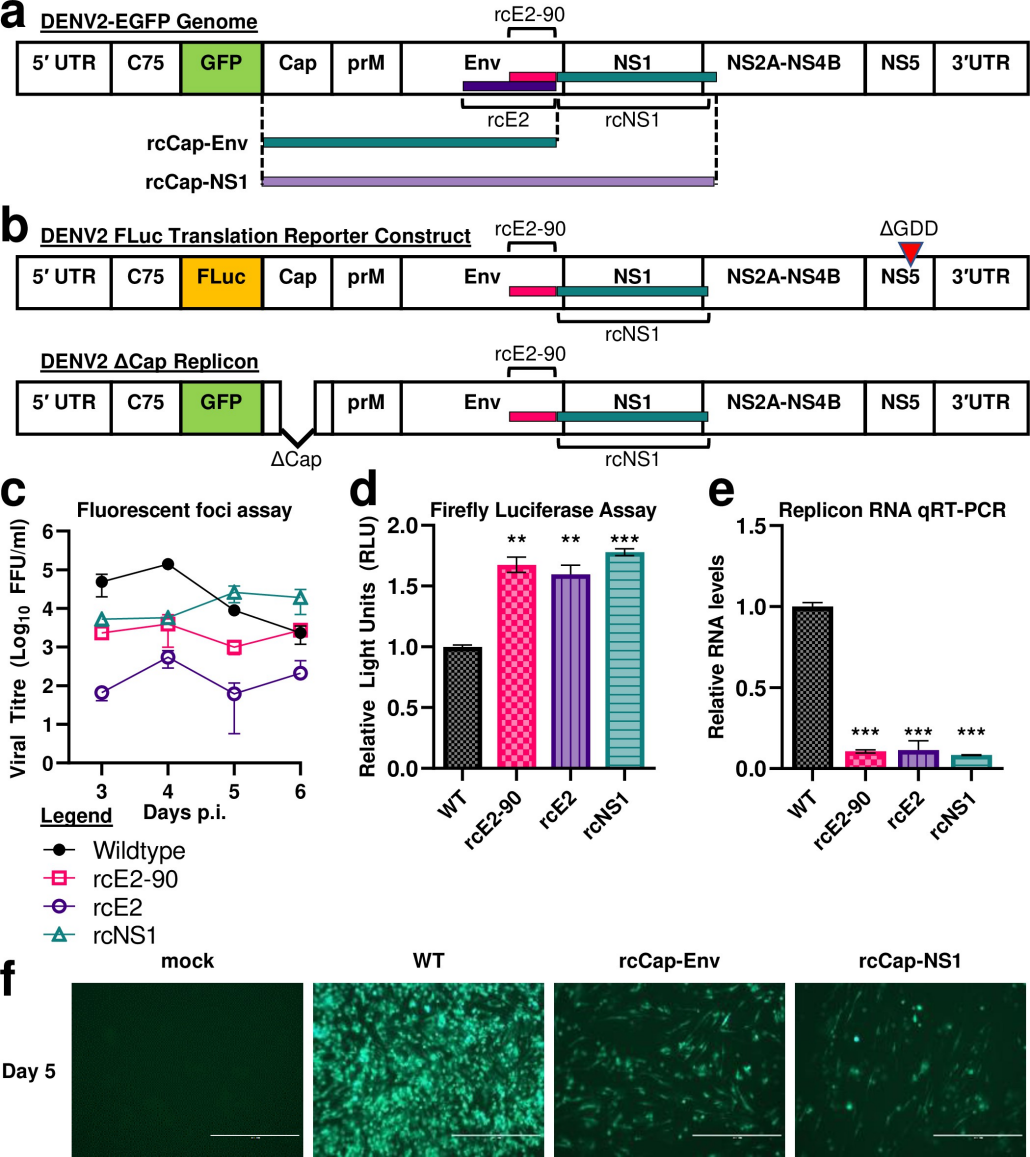
Characterisation of DENV2 genome recoding. **(a)** Genomic maps showing regions of the DENV2-EGFP genome recoded with silent mutations. DENV2-EGFP is a dengue reporter virus that expresses EGFP. **(b)** Genomic maps of DENV2 FLuc translation reporter construct and subgenomic replicon. DENV2 FLuc translation reporter construct contains a deletion of the NS5 protein GDD catalytic triad. DENV2 ΔCap Replicon is a subgenomic DENV2 replicon contains a deletion in the capsid coding region. **(c)** Viral growth kinetics of wildtype (non-recoded) and recoded DENV2-EGFP in BHK-21 cells. Viral titres were measured using fluorescent focus formation assay. FFU: fluorescent focus forming units. **(d)** Firefly luciferase assay was used to measure viral protein translation efficiency of non-recoded and recoded DENV2 FLuc constructs. **(e)** qRT-PCR was used to measure replicon RNA levels to compare the replicon RNA replication efficiency of non-recoded and recoded DENV2 replicons. **: p-value of <0.01. ***: p-value of <0.001. **(f)** Fluorescent microscopy analysis of BHK-21 cells infected with recoded DENV2-GFP (10x magnification) at day 5 post DNA-launch. Green fluorescent signal indicates DENV2-GFP infected cells. Mock: mock infected control cells. WT: cells infected with wildtype (non-recoded) DENV2-GFP.

We started by investigating the effects of virus genome recoding on DENV2-EGFP, which expresses EGFP as a reporter protein [41]. We constructed the rcE2-90 and rcE2 clones (**Fig 1A and Table 1**), with the respective recoded regions corresponding to codons 381 to 470 and codons 201 to 470 of the envelope protein (Env) coding region. We also constructed the rcNS1 clone, with the recoded region corresponding to the last 25 codons of the Env coding region, all of the NS1 coding region, and the first 25 codons of the NS2A coding region (402 codons total) (**Fig 1A and Table 1**).

**Table 1:** Detailed description of recoded DENV2 and ZIKV clones. The regions of the genome that were codon optimised are described in detail. Total number of codons that lie within these regions, as well as the number of codons that were mutated with silent mutations are also listed. The percentage of affected codons is calculated from the region targeted for recoding.

Virus	Clone	Recoded region of genome	Total no. of Codons	No. of mutated Codons	% of affected codons
DENV2	rcE2	Codons 201 to 470 of Env.	270	172	63.70%
DENV2	rcE2-90	Codons 381 to 470 of Env.	90	56	62.22%
DENV2	rcE2-60	Codons 411 to 470 of Env.	60	39	65%
DENV2	rcE2-50	Codons 421 to 470 of Env.	50	32	64%
DENV2	rcE2-40	Codons 431 to 470 of Env.	40	27	67.5%
DENV2	rcNS1	From the last 25 codons of Env to the first 25 codons of NS2A.	402	252	62.69%
DENV2	rcCap-prM	From the 26th codon of Capsid to the first 25 codons of Env.	279	165	59.14%
DENV2	rcCap-Env	From the 26th codon of Capsid to the 470th codon of Env, last 25 codons of Env remain untouched.	725	445	61.38%
DENV2	rcCap-NS1	From the 26th codon of Capsid to the first 25 codons of NS2A.	1127	697	61.85%
ZIKV	rcprM-NS3	From the last 25 codons of Capsid to the first 218 codons of NS3. Stops at a BsiWI restriction site.	1623	951	58.59%
ZIKV	rcprM-NS5	From the last 25 codons of Capsid to the first 126 codons of NS5. Stops at an AflII restriction site.	2549	1474	57.83%
ZIKV	rcCap-NS3	From the 26^th^ codon of Capsid to the first 218 codons of NS3. Stops at a BsiWI restriction site.	1695	993	58.58%
ZIKV	rcCap-NS5	From the 26^th^ codon of Capsid to the first 126 codons of NS5. Stops at an AflII restriction site.	2621	1516	57.84%

We DNA-launched these recoded DENV2-EGFP clones and wildtype (non-recoded) DENV2-EGFP in BHK-21 cells to investigate the effects of virus genome recoding. Compared to non-recoded DENV2-EGFP, the recoded rcE2, rcE2-90, and rcNS1 clones all demonstrated reduced replication efficiency (**Fig 1C**). This was correlated with a slower cell to cell spread by the recoded DENV2-EGFP clones (**S1B and S1C Fig**). This demonstrates that virus genome recoding by codon optimisation can reduce DENV2 replication efficiency.

### Recoded clones have higher protein expression efficiency but lower RNA replication efficiency

Next, we investigated if DENV2 genome recoding affects viral protein expression or viral RNA replication. A translation reporter construct was used to investigate viral protein expression efficiency, while a subgenomic replicon was used to investigate RNA replication efficiency (**Fig 1B**) [41]. We cloned the rcE2-90, rcE2, and rcNS1 mutations into these constructs (**Fig 1B**) and investigated their effects in BHK-21 cells. We found that the recoded rcE2-90, rcE2, and rcNS1 translation reporter constructs did have higher firefly luciferase activities compared to the non-recoded control (**Fig 1D**), while the rcE2-90, rcE2, and rcNS1 replicons had lower replicon RNA levels compared to the non-recoded control (**Fig 1E**). This indicates that DENV2 recoding results in a simultaneous enhancement of viral protein translation efficiency and reduction of viral RNA replication efficiency. This is consistent with the disruption of an RNA element that regulates the transition of the DENV2 RNA genome from the linear protein translation state to the competing circularised RNA replication state [37–40].

### The envelope stem coding region contains a putative RNA element

We wanted to determine if the phenotype of DENV2-EGFP-rcE2-90 requires the recoding of any specific region. We constructed three additional recoded clones, rcE2-60, rcE2-50, and rcE2-40, with the respective recoded regions narrowed down to 60, 50, and 40 codons respectively (**S2A Fig and Table 1**). We DNA-launched these recoded DENV2-EGFP clones in BHK-21 cells and used fluorescent microscopy to compare their replication efficiency. The rcE2-60 and rcE2-50 recoded clones retained the reduced replication efficiency of the rcE2-90 clone, while the rcE2-40 clone replicated faster (**S2B Fig**). The rcE2-50 clone differs from the rcE2-40 clone by a region corresponding to nucleotides 2197 to 2226 of the DENV2 genome and codons 421 to 430 of the envelope protein coding region. Codons 421 to 430 encode for the envelope protein stem region [42]. In wildtype DENV2, nucleotides 2197 to 2226 are predicted to be part of a RNA hairpin structure (**S2C and S5 Figs**), and the recoding mutations are predicted to disrupt this RNA hairpin. Therefore, this RNA hairpin may be a RNA element that contributes to efficient DENV2 replication. We named this putative RNA element the envelope stem RNA element (ESRE).

### Degree of recoding is correlated with slower DENV2-EGFP replication

Next, we investigated the effects of increasing the degree of genome recoding in the DEVN2-EGFP clones by constructing the rcCap-Env and rcCap-NS1 clones (**Fig 1A and Table 1**). We then DNA-launched wildtype (non-recoded) DENV2-EGFP as well as the rcCap-Env and rcCap-NS1 clones in BHK-21 cells to compare their replication efficiency. Compared to non-recoded DENV2-EGFP, the recoded rcCap-Env and rcCap-NS1 clones all demonstrated a great reduction in replication efficiency, as indicated by great reduction in EGFP positive cells (**Fig 1F**). This demonstrates that increasing the degree of genome recoding can lead to a further decrease in virus replication efficiency.

### Recoded DENV2 clones have reduced replication efficiency

**Fig 2:**
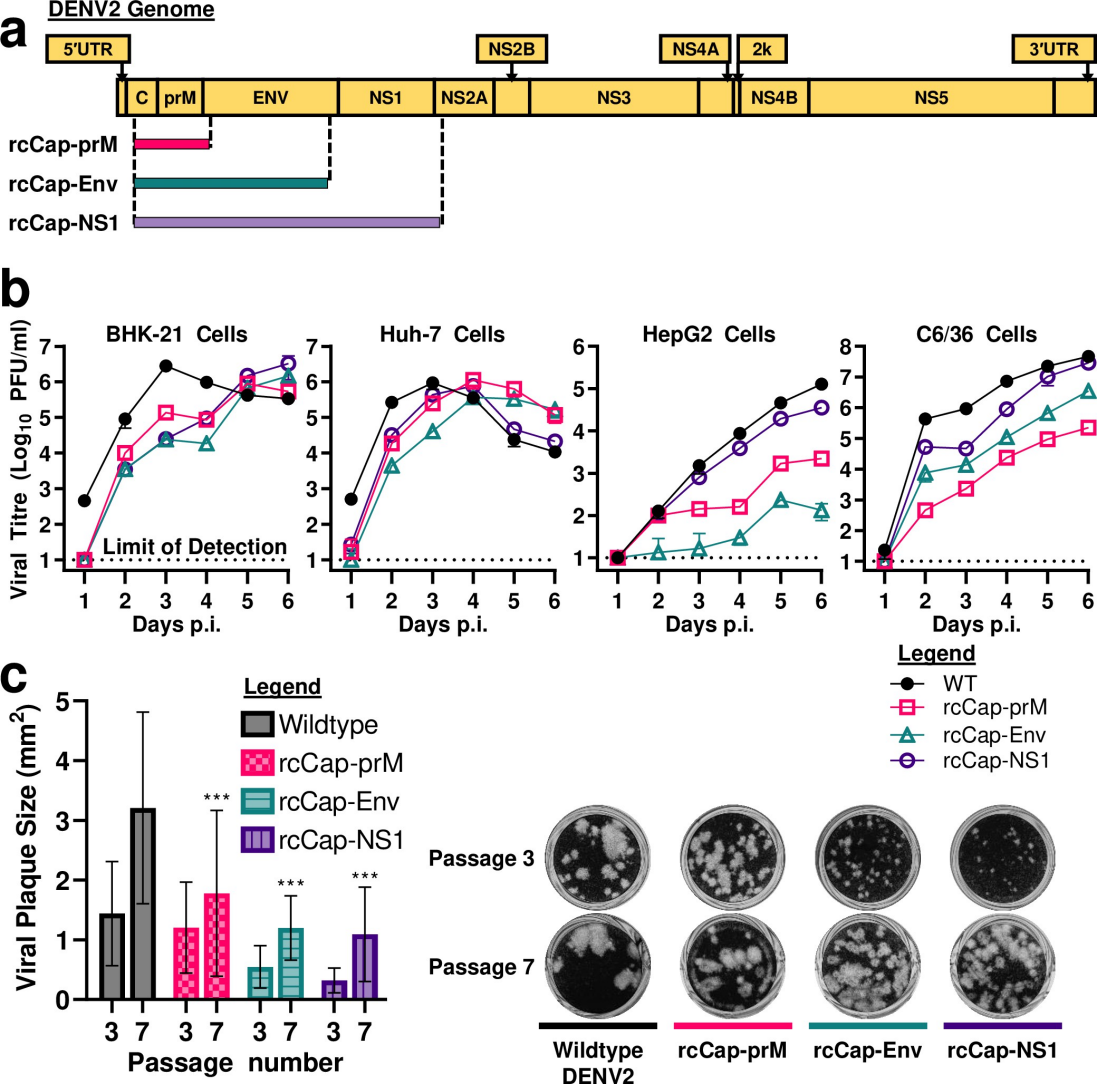
Recoded DENV2 clones have small plaque phenotype and reduced replication efficiency. **(a)** Genomic maps showing regions of the DENV2 genome recoded with silent mutations. **(b)** BHK-21 hamster kidney cells, Huh-7 human hepatocarcinoma cells, HepG2 hepatoma cells, and C6/36 Aedes mosquito cells were infected with wildtype or recoded DENV2 clones (rcCap-prM, rcCap-Env, and rcCap-NS1) at an MOI of 0.02. Viral titres were measured using plaque assay. Limit of detection for our plaque assay is 10 PFU/ml. **(c)** Plaque formation assay of wildtype and recoded DENV2 clones. Serial passage of wildtype and recoded DENV2 clones (rcCap-prM, rcCap-Env, and rcCap-NS1) in BHK-21 cells results in the emergence of large plaque mutants. Large variations in plaque size are due to the resulting mixed plaque population. Plaque sizes were measured in ImageJ using ViralPlaque Fiji macro. Statistical analysis of recoded virus plaque sizes at passage 7 was performed using the wildtype virus at passage 7 as a control. ***: p-value of <0.001.

We wanted to confirm that our results were not an experimental artifact of using an EGFP reporter virus. Therefore, we constructed three recoded DENV2 clones that are based on a wildtype DENV2-16681 backbone that does not carry any trans-genes. These clones were named rcCap-prM, rcCap-Env, and rcCap-NS1, which roughly corresponds to the regions in the genome that were targeted for recoding (**Fig 2A and Table 1**). The codon optimisation process introduced numerous silent mutations into the viral genome of these rcCap-prM, rcCap-Env, and rcCap-NS1 clones. **S3 Fig** shows an alignment that compares the recoded and non-recoded sequences from part of the prM and Env coding region. For any given region of recoding, approximately 57% to 61% of codons are mutated with silent mutations (**S3 Fig and Table 1**). The silent mutations alter the nucleotide sequence but not the encoded amino acid residue. These silent mutations may be a typical single nucleotide substitution at the third nucleotide of a codon. For example, the 89^th^ codon of the prM coding region is mutated from GAA to GAG, both of which code for Glutamic acid (**S3A Fig**). The silent mutations may also take the form of double or even triple nucleotide substitutions. For example, the 92nd codon of the prM coding region is mutated from TCA to AGC, both of which encode for Serine (**S3A Fig**).

The recoded DENV2 clones are derived from the same master sequence. For example, the recoded rcCap-NS1, rcCap-Env, and rcCap-prM clones share the exact same recoding mutations for the capsid and prM protein coding regions, an example of which is shown in **S3A Fig**. The recoded rcCap-NS1 and rcCap-Env clones also share the exact same recoding mutations for the Env protein coding region, an example of which can be seen in **S3B Fig**. Since the Env protein coding region of the rcCap-prM clone is not recoded, it shares the same Env protein coding sequence as wildtype DENV2 (**S3B Fig**). In other words, the rcCap-Env and rcCap-prM clones serve to narrow down the region of recoding seen in the rcCap-NS1 clone.

We compared the viral replication kinetics of wildtype and recoded DENV2 clones in BHK-21 hamster kidney cells, Huh-7 human hepatocarcinoma cells, HepG2 hepatoma cells, and C6/36 *Aedes* mosquito cells. The cells were inoculated at an MOI of 0.02 and plaque assay was used to compare viral titres. When compared to wildtype DENV2, the recoded DENV2 clones demonstrated delayed growth kinetics. In BHK-21, Huh-7, and HepG2 cells, the titres for the recoded DENV2 clones peaked at later timepoints. In HepG2 and C6/36 cells, the recoded DENV2 clones also replicated to lower viral titres (**Fig 2B and S1 Table**). The reduced replication efficiency in C6/36 mosquito cells is a favourable marker of attenuation that is correlated with reduced transmissibility by mosquitoes [27–30]. The rcCap-Env clone had the lowest replication efficiency in BHK-21, Huh-7, and HepG2 cells, and the second lowest replication efficiency in C6/36 cells. These results confirm that virus genome recoding by codon optimisation can reduce virus replication efficiency regardless of cell type or animal species.

### Genetic stability of wildtype and recoded DENV2

Next, we tested the genetic stability of the recoded DENV2 clones by serially passaging wildtype DENV2 and the recoded clones in BHK-21 cells. Initially, the recoded clones all had a small plaque phenotype compared to the wildtype DENV2 (**Fig 2C**). Serial passaging resulted in the emergence of a mixed plaque phenotype which resulted in a large variation in plaque sizes, with the large plaque mutants becoming dominant by passage 7 (**Fig 2C**). At passage 10, we performed next-generation sequencing on the wildtype DENV2, rcCap-Env, and rcCap-NS1 virus populations and identified potential rescue mutations and adaptation mutations, but no direct reversion mutations (**Tables 2 and 3**).

We identified the same a1522g (Env-M196V) and g1524a (Env-M196I) mutations in both the wildtype and recoded viruses. The a1522g and g1524a mutations had an exceedingly low co-occurrence frequency of 0.018%, indicating that once the virus acquired one of the mutations there was no more selection pressure to acquire the other. The Env-M196V mutation has been previously reported when wildtype DENV2 was similarly passaged in BHK-21 cells [43]. Therefore, we hypothesised that the Env-M196V/I mutations are BHK-21 cell line adaptations.

We also identified potential rescue mutations in the 5′UTR and in the capsid coding region (**Table 2**). We identified the same u71g nucleotide substitution mutation in the 5′UTR of the rcCap-Env and rcCap-NS1 virus population, but these occurred at low frequencies of 14.2% and 28% respectively. More importantly, we also identified a158u, u173c, and a192u nucleotide substitution mutations in the capsid coding region of the rcCap-Env virus population, as well as a158u, c181u, and a192u mutations in the rcCap-Env virus population (**Tables 2 and 3**). The a158u and a192u nucleotide substitutions had negligible co-occurrence frequencies of 0.07% or less in both recoded virus populations, meaning that there was selection pressure for one or the other, but not both (**Table 3**). The c181u mutation is silent, while the a158u, u173c, and a192u mutations result in capsid protein amino acid substitutions N21I, V26A, and R32S respectively (**Table 2**).

**Table 2:** Analysis of next-generation sequencing of wildtype and recoded DENV2 after serial passage. Relative frequencies of mutations are shown for each nucleotide position.

Nucleotide Position	Region of Genome	Nucleotide substitution	Amino acid substitution	Frequency in virus		
				Wildtype	rcCap-Env	rcCap-NS1
71	5′UTR	u to g	NA	0%	14.2%	28%
158	Capsid	a to u	Asn to Ile	0%	60.7%	21.6%
173	Capsid	u to c	Val to Ala	0%	52.5%	0%
181	Capsid	c to u	Leu to Leu	0%	0%	76%
192	Capsid	a to u	Arg to Ser	0%	39.4%	79.3%
1522	Env	a to g	Met to Val	31.2%	57.4%	22.1%
1524	Env	g to a	Met to Ile	37.4%	0%	58.4%

**Table 3:** Frequencies of co-occurrence of rescue mutations in the capsid coding region of (a) rcCap-Env and (b) rcCap-NS1.Nuleotides in bold and underscored: rescue mutation.

		Nucleotide							
(a) Nucleotide position in rcCap-Env	158	a	a	a	a	**u**	**u**	**u**	**u**
	173	u	**c**	u	**c**	u	**c**	u	**c**
	192	a	a	**u**	**u**	a	a	**u**	**u**
Co-occurrence frequency (%)		0	0.04	0.02	38.6	0.01	61.3	0	0.01
		Nucleotide							
(b) Nucleotide position in rcCap-NS1	158	a	a	a	a	**u**	**u**	**u**	**u**
	181	c	**u**	c	**u**	c	**u**	c	**u**
	192	a	a	**u**	**u**	a	a	**u**	**u**
Co-occurrence frequency (%)		0.03	0.03	3.44	75.4	20.7	0.3	0.04	0.03

### Predicted structure of DENV2 DCS-PK with recoding and rescue mutations

**Fig 3:**
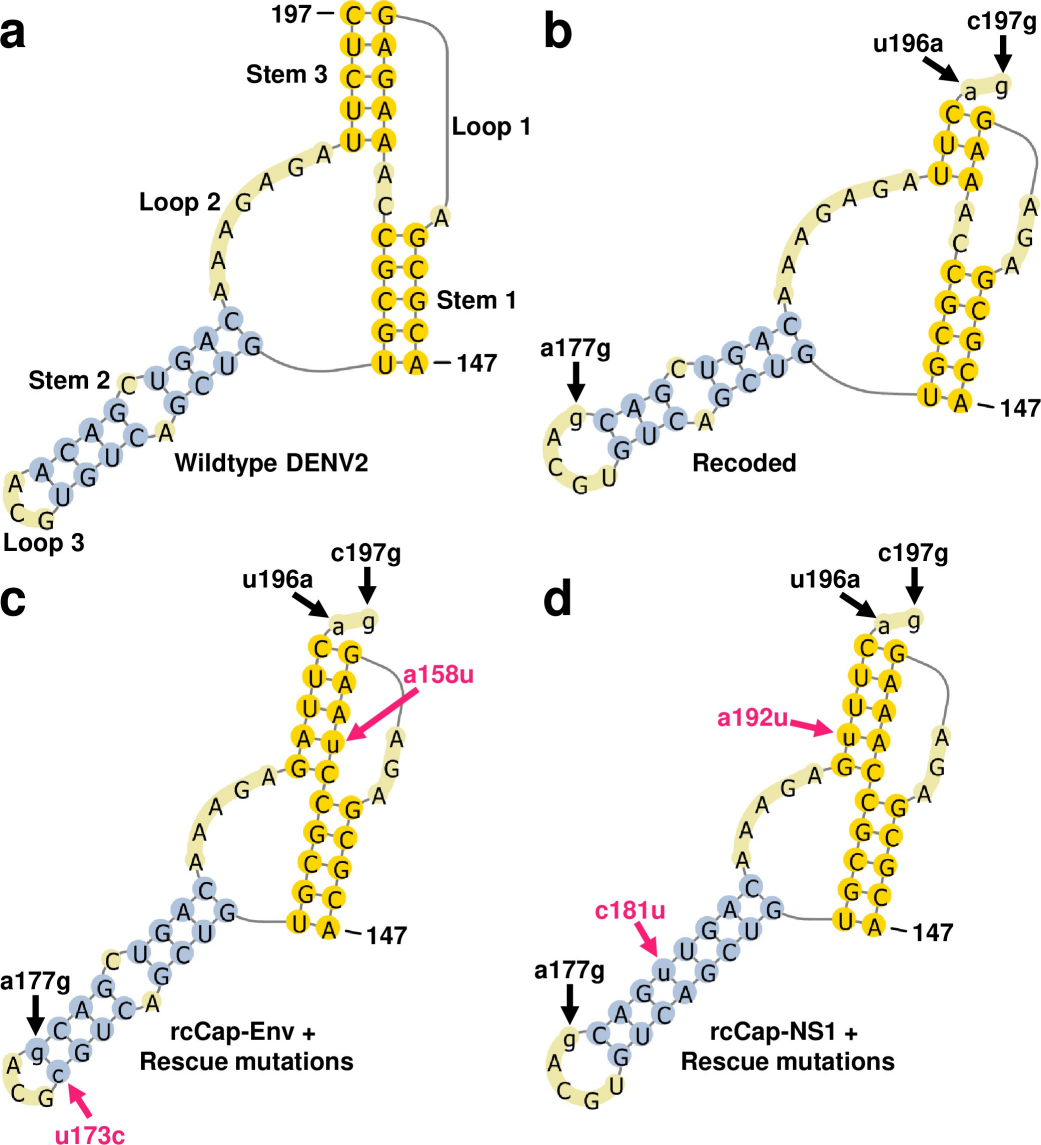
Predicted structure of DCS-PK RNA element in wildtype and recoded DENV2 clones. Capital letters indicate the original nucleotide sequence. Small letters indicate nucleotide substitutions. Black arrows indicate the positions of silent mutations introduced during codon optimisation: a177g in stem 2, as well as u196a and c197g in stem 3. **(a)** Predicted structure for Wildtype DENV2. **(b)** Predicted structure for recoded DENV2 clones (rcCap-prM, rcCap-Env, and rcCap-NS1). **(c) & (d)** Predicted structure of recoded DENV2 DCS-PK with rescue mutations acquired during serial passage. **(c)** Recoded rcCap-Env virus with the predominant a158u and u173c rescue mutations (indicated by the red arrows). **(d)** Recoded rcCap-NS1 virus with the predominant a158u and c181u rescue mutations (indicated by the red arrows).

The DENV2-rcCap-prM, rcCap-Env, and rcCap-NS1 clones all have the same recoded capsid coding sequence. This includes three nucleotide substitutions that can be mapped to the previously reported DCS-PK element: a177g, u196a, and c197g. The rescue mutations at nucleotide positions 158, 173, 181, and 192 can also be mapped to the DCS-PK. Therefore, we performed *in silico* modelling to investigate the effects of the recoding and rescue mutations on the DCS-PK [32,44–46]. The a177g mutation in stem 2 as well as the u196a and c197g mutations in stem 3 were found to disrupt the structure of the DCS-PK in recoded DENV2 (**Fig 3A and 3B**). The a158u and a192u rescue mutations are predicted to base pair with a192 and a158 respectively, and these interactions are predicted to restore stem 3 of the DCS-PK (**Fig 3C and 3D**). The a158u and a192u rescue mutations cannot base pair with each other, which would explain their negligible co-occurrence frequency (**Table 3**). The u173c and c181u are also predicted to create additional base pairings within the DCS-PK stem 2 (**Fig 3C and 3D**). The u173c mutation introduces a base pairing with a177g, which is one of the original recoding mutations, while the c181u mutation introduces a base pairing with a169. Therefore, the capsid rescue mutations are gain-of-function mutations that create additional base pairings within the DCS-PK.

### Constructing recapitulatory rescue clones of recoded DENV2

We cloned various combinations of the rescue mutations into the rcCap-Env infectious clone (**S4 Fig**). We chose the rcCap-Env clone for our downstream experiments because it showed the lowest replication efficiency in human and mammalian cells. The rcCap-Env+rsCE rescue clone contains the a158u (Cap-N21I), u173c (V26A), and a1522g (Env-M196V) mutations and recapitulates the dominant species of the rcCap-Env virus population at passage 10. We also constructed two additional rescue clones to deconvolute the contributions of the capsid and envelope rescue mutations: rcCap-Env+rsCap contains the a158u (Cap-N21I) and u173c (Cap-V26A) mutation while rcCap-Env+rsEnv contains only the a1522g (Env-M196V) mutation. As a control, we cloned the a1522g (Env-M196V) mutation into wildtype DENV2 to construct WT+rsEnv. WT+rsEnv recapitulates the genotype of one of the dominant species of the wildtype virus population at passage 10.

All the rescue clones were viable as they were able to form plaques (**S5 Fig**). Amongst all the wildtype, recoded, and rescue clones, the WT+rsEnv rescue clone that possesses the Env-M196V mutation forms the largest plaques (**S5 Fig**). In contrast, the rcCap-Env+rsEnv rescue clone that also possesses the Env-M196V forms the smallest plaques.

### RNA SHAPE analysis shows loss of RNA structures in Recoded DENV2

We wanted to confirm if DENV2 genome recoding could disrupt genomic RNA structures, and if the disruption remained stable after serial passaging. Therefore, we performed RNA SHAPE-MaP analysis on wildtype DENV2 and the rcCap-Env+rsCap rescue clone [47]. Using the results of our SHAPE analysis as a constraint, we also performed *in silico* modelling of wildtype and recoded virus RNA structural elements [48,49].

**Fig 4:**
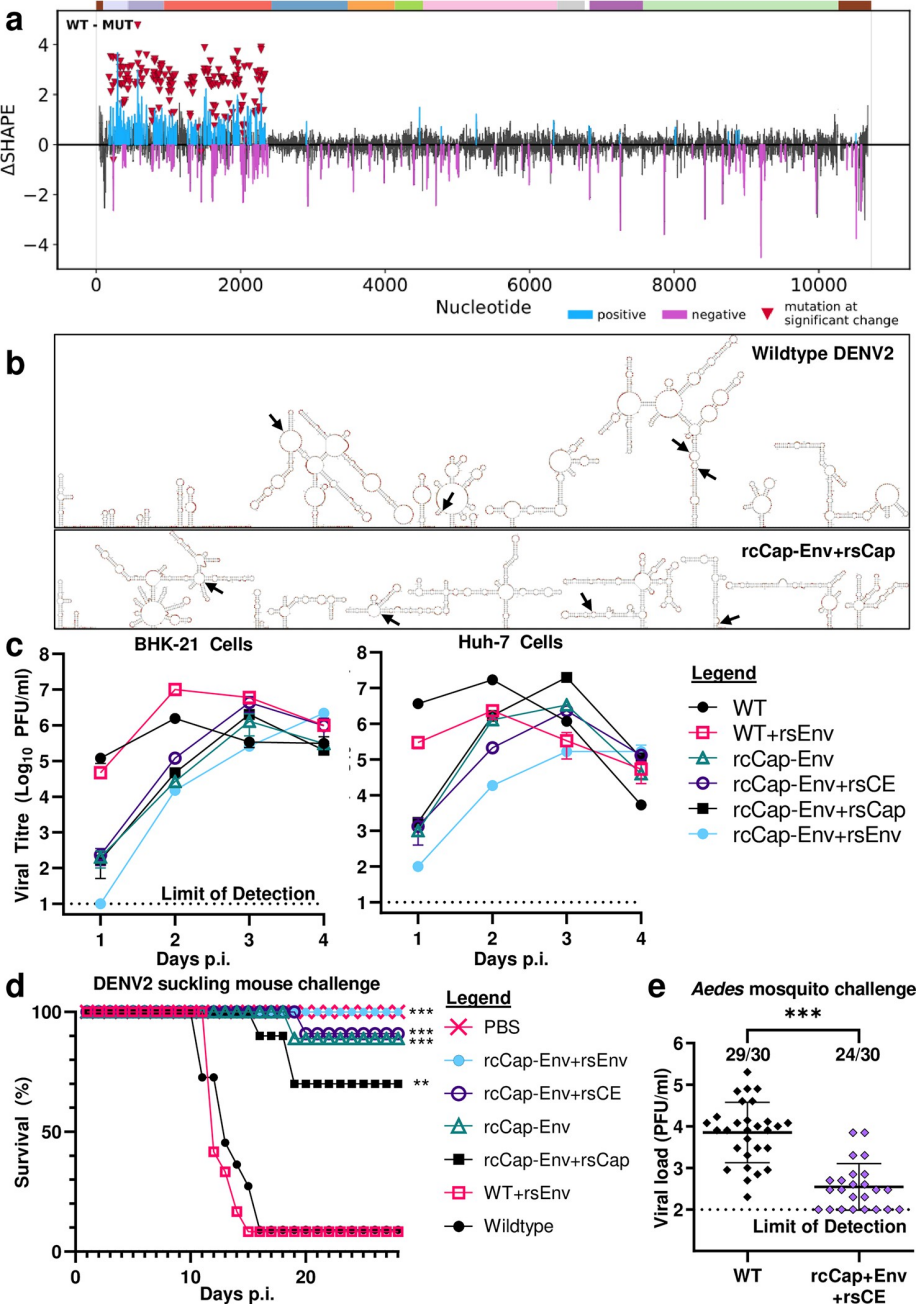
Recoded DENV2 has disrupted RNA structural elements and genetically stable attenuation. **(a)** RNA SHAPE-Map analysis of DENV2 genomic RNA structures. Analysis was performed on BHK-21 cells infected with either wildtype DENV2 or the rcCap-Env+rsCap recoded rescue clone. After infection, NAI treatment was performed to modify single-stranded nucleotides, after which total RNA extraction, cDNA library preparation and Illumina sequencing was performed according to the SHAPE-Map protocol. Differences in SHAPE were assessed using ΔSHAPE. Inverted red triangle indicates location of mutations found in the rcCap-Env+rsCE clone. Positive and negative changes to reactivity compared to wildtype DENV2 are indicated in blue and purple respectively. Positive changes represent reduced reactivity, indicating increased base pairing by a particular nucleotide. **(b)** Predicted RNA secondary structures in the 5′UTR and structural protein coding region of wildtype DENV2-16681 and rcCap-Env+rsCap rescue clone, corresponding to nucleotides 1 to 2421 of the DENV2 genome. *In silico* modelling of DENV2 genomic RNA secondary structures was performed using the Superfold pipeline with RNAstructure v6.3 as the backend, with the results of our SHAPE analysis incorporated as a constraint. RNA structures were then visualized using VARNA 3.93 and a custom script to map SHAPE reactivity data onto the resulting figure. Black arrows indicate nucleotide positions 500, 1000, 1500, and 2000 of the respective DENV2 genomes. **(c)** Rescue clones of recoded DENV2-rcCap-Env retain attenuated growth kinetics. BHK-21 hamster kidney cells and Huh-7 human hepatocarcinoma cells were infected with wildtype DENV2, wildtype DENV2 with Env-M196V cell line adaptation mutation (WT+rsEnv), recoded DENV2 (rcCap-Env), and rescue mutants of DENV2-rcCap-Env (rcCap-Env+rsCE, +rsCap, and +rsEnv) at an MOI of 0.1. Viral titres were measured using plaque assay. Limit of detection for our plaque assay is 10 PFU/ml. **(d)** Recoded and rescue clones of DENV2 demonstrate attenuation of neurovirulence in suckling mice. Newborn outbred white ICR mice that were less than 24 hours old were challenged by intracranial inoculation with wildtype DENV2, WT+rsEnv, rcCap-Env, or rc+rsCE clones at a dose of 10^2 PFU per mouse. The mice were kept for four weeks and observed daily for clinical symptoms. Mice that reached a humane endpoint were euthanized. Group sizes: PBS control, n = 10; wildtype DENV2, n = 11; WT+rsEnv, n = 12; rcCap-Env, n = 9; rCap-Env+rsCE clone, n = 11; rcCap-Env+rsCap, n = 10; rcCap-Env+rsEnv, n = 9. **: p-value of <0.01. ***: p-value of <0.001. **(e)** Recoded DENV2 demonstrates attenuation in its *Aedes albopictus* mosquito vector. *Aedes albopictus* mosquitoes were fed an infectious blood meal containing 2.5 x 10^7^ PFU/ml of either wildtype DENV2 or DENV2-rcCap-Env+rsCE. At 11 days post challenge, the mosquitoes were harvested and their infection status and viral loads were determined using plaque assay. Group sizes for both were n = 30. Limit of detection is 100 PFU/ml. ***: p-value of <0.001.

We found that almost all the RNA structural elements that are found in the structural protein coding region of wildtype DENV2 are disrupted and no longer found in recoded DENV2 (**Fig 4A, 4B, see S6 and S7 for high resolution figures**). Furthermore, we found that the a158u (Cap-N21I) and u173c (Cap-V26A) rescue mutations did not play any meaningful role in reversing the large-scale disruptions to RNA structural elements. This confirms that DENV2 genome recoding by codon optimisation results in the disruption of potential RNA elements in the virus genome. It also indicates that these disruptions are genetically stable.

### Recapitulatory rescue clones of recoded DENV2 retain delayed viral growth kinetics

Next, we compared the viral replication kinetics of rcCap-Env and the derivative rescue clones in BHK-21 cells and Huh-7 cells. The cells were inoculated at an MOI of 0.1 and plaque assay was used to compare viral titres. We found that the rescue mutations affect virus particle production, but do not rescue the delayed replication kinetics of recoded DENV2. In both BHK-21 and Huh-7 cells, the rsCap-Env+rsCE, +rsCap, and +rsEnv rescue clones all retained the delayed peak titre of parental rsCap-Env (**Fig 4C and S2 Table**). The addition of the a158u (Cap-N21I) and u173c (Cap-V26A) rescue mutations to the rcCap-Env backbone allows the resulting rcCap-Env+rsCap rescue clone to achieve higher peak titres in both BHK-21 and Huh-7 cells (**Fig 4C and S2 Table**). The a1522g (Env-M196V) rescue mutation acts as a more specific BHK-21 cell line adaptation; the addition of the a1522g (Env-M196V) mutation to the wildtype DENV2, rcCap-Env, and rcCap-Env+rsCap clones confers higher peak titres on the resulting WT+rsEnv, rcCap-Env+rsEnv, and rcCap-Env+rsCE clones respectively, but only in BHK-21 cells (**Fig 4C and S2 Table**). The opposite was true in Huh-7 cells, with the Env-M196V conferring lower peak titres (**Fig 4C and S2 Table**).

Curiously enough, the addition of the a1522g (Env-M196V) mutation to the rcCap-Env backbone confers a further delay in replication kinetics on the resulting rcCap-Env+rsEnv rescue clone (**Fig 4C and S2 Table**). This effect was not observed in the rcCap-Env+rsCE rescue clone with the additional capsid rescue mutations or in the wildtype backbone. Therefore, the effect of the a1522g (Env-M196V) mutation depends on whether it is cloned into a wildtype backbone or into some specific recoded backbone. This makes sense when we consider that the underlying a1522g RNA mutation can have its own effects at the functional RNA level.

### Recoded DENV2 demonstrates genetically stable attenuation in suckling mice

We investigated whether recoded DENV2 demonstrates genetically stable *in vivo* attenuation. We investigated *in vivo* attenuation using a suckling mouse model of neurovirulence as it is a well-established model for studying flavivirus attenuation [27–29]. Newborn outbred white ICR mice that were less than 24 hours old were inoculated intracranially at a dose of 10^5 PFU/ml with wildtype, recoded, or rescue clones. The mice were kept for four weeks post inoculation and observed daily for clinical symptoms and euthanised when they reached a humane endpoint [27,28].

Wildtype DENV2 and WT+rsEnv had similar lethality rates of 91% and 92% (n = 10/11 and 11/12) respectively (**Fig 4D**). In contrast, recoded rcCap-Env and its derivative rescue clones all demonstrated *in vivo* attenuation; the lethality rate of the rcCap-Env, +rsCE, +rsCap, and +rsEnv was significantly lower at 11%, 9%, 30%, and 0% respectively (n = 1/9, 1/11, 3/10, and 0/9) (**Fig 4D**). This demonstrates that rcCap-Env possesses genetically stable attenuation, as serial passaging does not result in mutations that can restore virulence.

### Recoded DENV2 demonstrates attenuation in *Aedes albopictus* mosquitoes

Next, we investigated if recoded DENV2 is attenuated in its *Aedes* mosquito vector [30]. This is a blood meal challenge model, where mosquitoes are fed an infectious blood meal containing DENV2. The mosquitoes are then kept for 11 days after oral infection, after which we use plaque assay to determine their infection status and viral load. We typically challenge our mosquitoes with a blood meal containing DENV2 at a concentration of 5 x 10^6^ PFU/ml, as this concentration is sufficient to infect mosquitoes with wildtype DENV2. However, when we attempted to challenge mosquitoes with recoded virus at the same virus concentration of 5 x 10^6^ PFU/ml, none of the recoded viruses could establish an infection. Therefore, we repeated the mosquito challenge with the virus at a 5-fold higher concentration of 2.5 x 10^7^ PFU/ml. While most of the recoded clones are unable to replicate to such a high titre, the recoded rcCap-Env+rsCE clone replicates well enough in BHK-21 cells to reach this titre.

Therefore, we were able to challenge *Aedes albopictus* mosquitoes with an infectious blood meal containing 2.5 x 10^7^ PFU/ml of either wildtype DENV2 or recoded DENV2-rcCap-Env+rsCE. The mosquitoes were kept for 11 days after oral infection, after which we used plaque assay to determine their infection status and viral load. Compared to wildtype DENV2, recoded DENV2-rcCap-Env+rsCE was attenuated in *Aedes albopictus* mosquitoes. Wildtype virus was able to establish infection in 29/30 mosquitoes, whereas recoded DENV2-rcCap-Env+rsCE was only able to only infected 24/30 mosquitoes (**Fig 4E**). Furthermore, mosquitoes that were infected with recoded DENV2-rcCap-Env+rsCE were also found to carry a lower viral load (**Fig 4E**). This demonstrates that recoded DENV2 has *in vivo* attenuation in both mice and mosquitoes.

### Recoded ZIKV demonstrates reduced replication efficiency and *in vivo* attenuation

**Fig 5:**
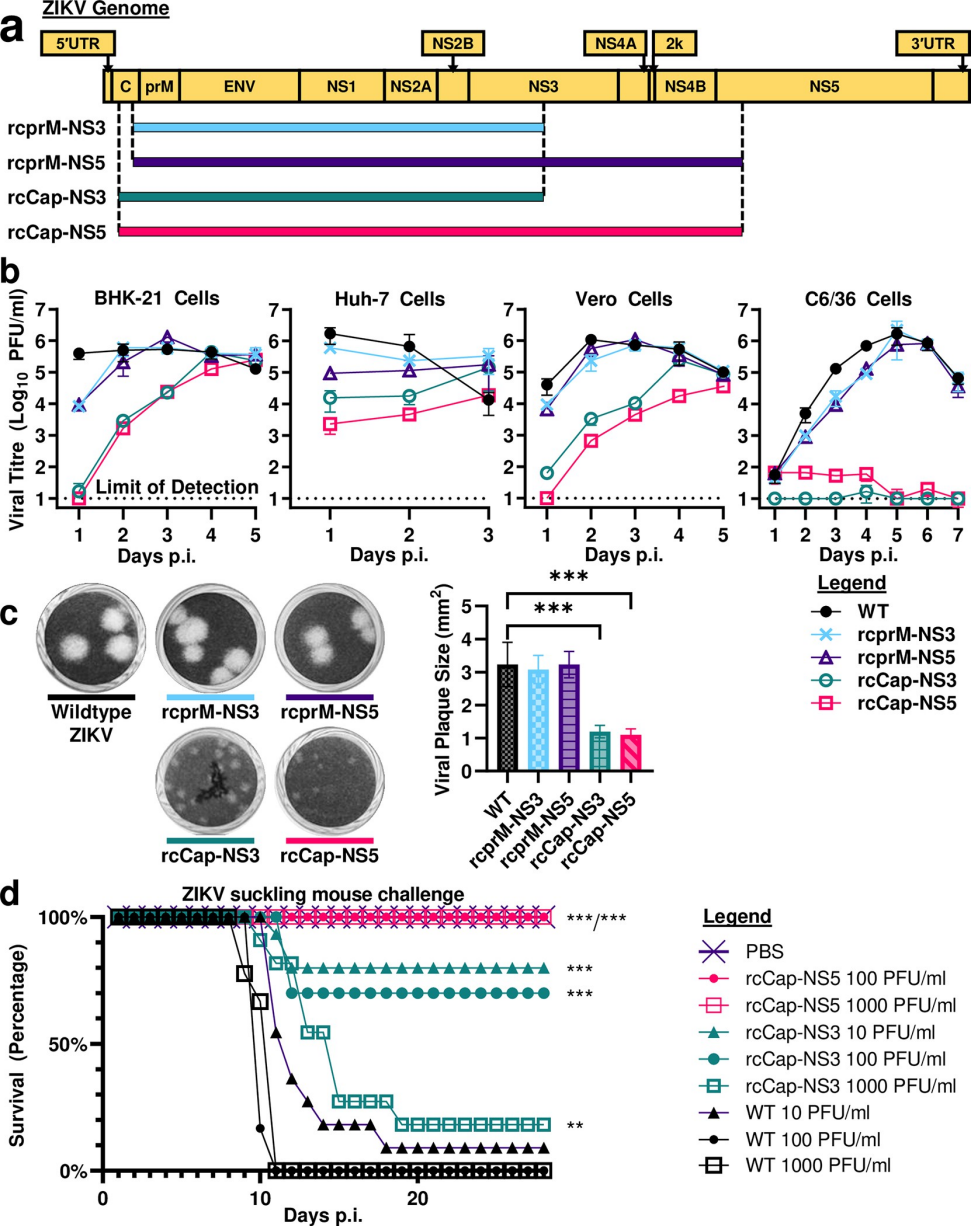
Recoded ZIKV clones are attenuated. **(a)** Genomic maps showing regions of the ZIKV genome recoded with silent mutations. **(b)** Recoded ZIKV clones are viable. Recoding of the capsid coding region confers a small plaque phenotype. ***: p-value of <0.001. **(c)** Recoded ZIKV clones have reduced replication efficiency. BHK-21 hamster kidney cells, Huh-7 human hepatocarcinoma cells, Vero monkey cells, and C6/36 Aedes mosquito cells were infected with wildtype or recoded ZIKV clones (rcprM-NS3, rcprM-NS5, rcCap-NS3, and rcCap-NS5) at an MOI of 0.01. Viral titres were measured using plaque assay. Limit of detection for our plaque assay is 10 PFU/ml. **(d)** Recoded ZIKV clones demonstrate attenuation of neurovirulence in suckling mice. Newborn outbred white ICR mice that were less than 24 hours old were challenged by intracranial inoculation with wildtype ZIKV, ZIKV-rcCap-NS3, or ZIKV-rcCap-NS5. The mice were kept for four weeks and observed daily for clinical symptoms. Mice that reached a humane endpoint were euthanized. Group sizes: PBS control, n = 10; wildtype ZIKV 10^1^ PFU/ml, n = 11; wildtype ZIKV 10^2^ PFU/ml, n = 12; wildtype ZIKV 10^3^ PFU/ml, n = 11; rcCap-NS3 10^1^ PFU/ml, n = 15; rcCap-NS3 10^2^ PFU/ml, n = 10; rcCap-NS3 10^3^ PFU/ml, n = 11; rcCap-NS5 10^2^ PFU/ml, n = 12; rcCap-NS5 10^3^ PFU/ml, n = 14.

Finally, we investigated whether genome recoding can attenuate another flavivirus. We recoded a ZIKV infectious clone that we have previously constructed [27]. The resulting clones are ZIKV-rcprM-NS3, rcprM-NS5, rcCap-NS3, and rcCap-NS5, where the name of the clone corresponds to the regions targeted for recoding (**Fig 5A and Table 1**).

We compared the viral replication kinetics of wildtype and recoded ZIKV clones in BHK-21 hamster kidney cells, Huh-7 human hepatocarcinoma cells, Vero monkey cells, and C6/36 *Aedes* mosquito cells. The cells were inoculated at an MOI of 0.01 and plaque assay was used to compare viral titres. When compared to the wildtype virus, the recoded ZIKV clones demonstrated reduced replication efficiency in mammalian, human, and mosquito cells. In general, recoded ZIKV clones replicated to lower peak virus titres, and their viral titres peaked at later timepoints (**Fig 5B and S3 Table**). Taking the ZIKV-rcprM-NS3 clone as the baseline, recoding of the prM to NS3 coding regions in the ZIKV-rcprM-NS3 clone conferred a mild reduction in virus replication efficiency. Thereafter, increasing the degree of recoding resulted in further reductions in virus replication efficiency. The degree of reduction depended on which region was the target of further recoding: the NS3 to NS5 coding region had the smallest effect, the capsid coding region by itself had a greater effect, and targeting both the capsid and NS3 to NS5 coding regions had the greatest effect (**Fig 5B and S3 Table**). Both the ZIKV-rcCap-NS3 and rcCap-NS5 have near lethal phenotypes in C6/36 mosquito cells (**Fig 5B and S3 Table**). This indicates that recoding the ZIKV capsid coding region disrupted an RNA element that is critical for replication in mosquito cells. We also found that recoding the capsid coding region conferred a small plaque phenotype, which is consistent with the greater reduction in replication efficiency (**Fig 5C**).

Finally, we investigated the in vivo attenuation of the ZIKV-rcCap-NS3 and ZIKV-rcCap-NS5 clones using our suckling mouse model [27]. Newborn outbred white ICR mice that were less than 24 hours old were inoculated intracranially at a dose of 10^1^, 10^2^, or 10^3^ PFU/ml with wildtype or recoded ZIKV. Wildtype ZIKV demonstrated a high degree of virulence at doses of 10^1^, 10^2^, and 10^3^ PFU/ml, with lethality rates of 90.9%, 100%, and 100% respectively (n = 1/11, 0/12, and 0/11) (**Fig 5D**). In contrast, ZIKV-rcCap-NS3 demonstrated in vivo attenuation for the same three doses, with lower lethality rates of 20%, 30%, and 82% respectively (n = 12/15, 7/10, and 9/11) (Fig 4E). The ZIKV-rcCap-NS5 clone demonstrated an even higher degree of attenuation: at the higher doses of 10^2^ or 10^3^ PFU/ml it had lethality rates of 0% (n = 12/12 and 14/14) (**Fig 5D**). This indicates that a greater degree of recoding is correlated with a greater degree of attenuation. This also demonstrates that our recoding approach can attenuate both DENV2 and ZIKV.

## Discussion

In this study, we demonstrate a virus genome recoding approach that targets the functional RNA elements in the flavivirus genome. These functional RNA elements are essential for efficient virus replication [26,31–40]. We hypothesised that genome recoding via codon optimising of the flavivirus protein coding region will introduce silent mutations that disrupt the sequence and function of these RNA elements, leading to a reduction in viral replication efficiency. Our study confirms that this flavivirus recoding approach translates into reduced replication efficiency in mammalian, human, and mosquito cells as well as *in vivo* attenuation in both mice and mosquitoes.

Our approach of virus genome recoding by codon optimisation offers several advantages over prior flavivirus genome recoding approaches. These prior studies on DENV2 and ZIKV performed genome recoding by codon deoptimisating [13,19,22]. This codon deoptimisation altered the protein coding region of the viral genome such that the codon pair frequency was no longer favourable for human cells, but still favourable for mosquito cells [13,19,22]. The disadvantage of these codon deoptimisation approaches is that the mechanism of attenuation is highly dependent on animal species [13,19,22]. For example, codon deoptimised DENV2 demonstrated attenuation in LLC-MK2 monkey cells, but did not demonstrate any attenuation in BHK-21 hamster cells or C6/36 mosquito cells [13]. Similarly, codon deoptimised ZIKV demonstrated attenuation in Vero monkey cells, but not C6/36 mosquito cells [22]. Furthermore, codon deoptimised DENV2 did not demonstrate any in vivo attenuation in *Aedes aegypti* mosquitoes [19]. This lack of attenuation in mosquitoes is undesirable for a flavivirus vaccine because the vaccine strain should be attenuated in its mosquito vector to prevent vaccine transmission [28]. In contrast to these prior codon deoptimisation approaches, our codon optimisation approach operates by an attenuation mechanism that functions regardless of cell type or animal species. Therefore, our recoded DENV2 and recoded ZIKV clones demonstrate attenuation in human, mammalian, and mosquito cells. Most importantly, our recoded DENV2 clone demonstrates *in vivo* attenuation in both mice and *Aedes* mosquitoes.

We were able to demonstrate that the recoded DENV2-rcCap-Env+rsCE clone is attenuated in *Aedes* mosquitoes. We note that the recoded DENV2-rcCap-Env+rsCE clone had a lower infection rate in *Aedes albopictus* mosquitoes: when we used an infectious blood meal with a virus concentration of 2.5 x 10^7^ PFU/ml, wildtype DENV2 could infect 29/30 mosquitoes, but DENV2-rcCap-Env+rsCE was only able to infect 24/30 mosquitoes (**Fig 4E**). We were unable to successfully infect mosquitoes with the other recoded clones, such as DENV2-rcCap-Env, DENV2-rcCap-Env+rsCap, and DENV2-rcCap-Env+rsEnv, at least when using an infectious blood meal with a lower virus concentration of 5 x 10^6^ PFU/ml. This indicates that the recoded DENV2 clones have reduced ability to establish an infection in *Aedes albopictus* mosquitoes. We hypothesise that our genome recoding was able to disrupt RNA elements that are important for replication in mosquito cells. It is even possible that some of these disrupted RNA elements could be required to recruit mosquito-specific host factors that are required replication in mosquito cells. This may raise the threshold required for recoded DENV2 to establish a successful *in vivo* infection in *Aedes* mosquitoes. In future, we could try to repeat our *Aedes* mosquito challenge studies to determine if the other recoded DENV2 clones require a higher challenge dose to establish an *in vivo* infection in *Aedes* mosquitoes, or if these other recoded DENV2 clones are unviable in *Aedes* mosquitoes. To do this, we may have to concentrate the viruses to achieve a sufficiently high virus concentration [27].

Our results also validate the mechanism of attenuation of our genome recoding approach. As expected, genome recoding resulted in disruption of flavivirus genome RNA structural elements (**Figs 4A, 4B, S5 and S6**). This disruption of RNA elements led to the enhancement of viral protein translation and reduction in viral RNA replication efficiency (**Fig 1D and 1E**). The enhancement of viral protein translation might seem unexpected. This is because the increase in protein translation should mean an increased production of the flavivirus NS5 protein that is responsible for viral RNA replication. However, prior studies on flavivirus RNA elements provides two potential explanations for this observation. The first explanation is that many flavivirus RNA elements are known to regulate both flavivirus RNA replication and flavivirus protein translation [26,31–40,50]. For example, RNA elements such as the SLA, UFS, 5’CS, 3’CS, 5’UAR, 3’UAR, cHP, and DCS-PK regulate the transition of the flavivirus genome from the linear conformation to a circular conformation [26,31–40, 50]. The initial state of the genome is the linear conformation, which is responsible for viral protein translation [39]. After viral protein translation has taken place, RNA elements help the genome to transition to the circular conformation, which shuts down protein translation and initiates viral RNA replication [39]. The second explanation is that flavivirus RNA elements are also known to recruit host factors that regulate both flavivirus RNA replication and flavivirus protein translation. For example, RNA elements in the flavivirus genome are known to recruit the host factors such as AUF1 and PABPC1 to regulate flavivirus RNA replication and flavivirus protein translation respectively [51,52].

Therefore, we hypothesise that our genome recoding disrupts the RNA elements that regulate the transition from the linear genome conformation to the circular conformation [26,31–40,50]. This means the genome is stuck in the initial protein translation state for an extended duration, which results in an initial enhancement of protein translation efficiency. However, genome transition to the circular RNA replication state is delayed, which results in a reduction in RNA replication efficiency. This reduction in RNA replication efficiency is an exponential reduction that affects all subsequent rounds of viral RNA replication. Therefore, the exponential reduction of RNA replication efficiency overcomes any increase in the initial protein translation efficiency, which results in an overall decrease in viral replication efficiency. An alternative hypothesis is that our genome recoding disrupts the RNA elements that are required to recruits host factors that regulate flavivirus RNA replication [52]. For example, the loss of RNA elements that recruit the host factor AUF1 would result in delayed initiation of flavivirus RNA replication [52]. A third hypothesis is that our flavivirus genome recoding approach introduces mutations that are more susceptible to activating host restriction factors and host innate immune responses [19,53]. For example, genome recoding in influenza A virus introduces increased CpG dinucleotide frequencies, which was found to induce stronger antiviral host immune responses [53]. All these hypotheses regarding the molecular mechanisms of our flavivirus genome recoding approach will need to be validated in future studies.

We have identified several questions that need to be addressed in our future studies. First, we will need to investigate our recoded viruses in an adult mouse model to confirm that our recoded viruses are both attenuated and immunogenic. The second question is how recoding different regions of the flavivirus genome can have different effects. Our current study found that recoding the ZIKV capsid coding region confers a relatively high degree of attenuation, especially in mosquito cells. We hypothesise that this loss of viability in mosquito cells is caused by the disruption of conserved RNA structural elements in the capsid coding region [34]. Therefore, a more targeted disruption of these RNA elements in the capsid coding region will be useful for future vaccine development. We also found that there is no additive effect when we simultaneously recoded both the DENV2 Env and NS1 coding regions, even though recoding either region by itself resulted in reduced replication efficiency. This implies some degree of redundancy, or even antagonism between the RNA elements in the DENV2 Env and NS1 coding regions. On the other hand, in ZIKV we found that increasing the degree of recoding by large increments does correlate with increased attenuation. The third question is to determine the molecular attenuation mechanism underlying our flavivirus genome recoding approach. For example, we could use a methodology called Sequencing of Psoralen crosslinked, Ligated, and Selected Hybrids (SPLASH) to determine if our flavivirus genome recoding can disrupt long range RNA-RNA interactions between RNA elements [34]. This would enable us to look at whether recoded viruses undergo reduced or enhanced viral genome circularisation. We could also perform RNA immunoprecipitation experiments to determine if our flavivirus genome recoding is disrupting or enhancing RNA element interaction with specific host cellular factors or host restriction factors [54]. Finally, we could investigate whether our recoded flavivirus clones are able to induce stronger host immune responses, both in cell culture and *in vivo* [53].

In conclusion, our flavivirus genome recoding approach targets the RNA elements that regulate RNA replication. This results in reduced viral replication efficiency in human, mammalian, and even mosquito cells as well as *in vivo* attenuation in a suckling mouse model and *Aedes albopictus* mosquito model. Serial passaging recoded DENV2 does not result in the emergence of mutations that can rescue the delayed replication kinetics or *in vivo* attenuation of recoded DENV2. This demonstrates that our recoding approach confers genetically stable attenuation. Therefore, our recoding approach has the potential to produce attenuated backbones for the development of next-generation flavivirus vaccines.

## Materials and methods

### Ethics statement

Mouse model studies were reviewed and approved by the National University of Singapore Institutional Animal Care and Use Committee (IACUC) under protocol number R18-0488.

### Cell culture and cell culture media

The following cell lines were used in this study: BHK-21 baby hamster kidney cells (ATCC CCL-10, USA), Huh-7 human hepatoma cells (kindly provided by Dr. Priscilla Yang, Stanford University, USA), HepG2 human hepatoma cells (ATCC HB-8065), Vero E6 African green monkey kidney cells (ATCC CRL- 1586), and C6/36 *Aedes albopictus* larvae cells (ATCC CRL-1660, USA). BHK-21 cells were cultured in Roswell Park Memorial Institute 1640 (RPMI) medium (Sigma-Aldrich) supplemented with 10% foetal calf serum (FCS) and 2g/L of NaHCO3. Huh-7, HepG2, and Vero cells were cultured in Dulbecco’s Modified Eagle’s medium (DMEM) (Sigma-Aldrich) supplemented with 10% FCS and 2g/L of NaHCO3. BHK-21, Huh-7, HepG2, and Vero cells were cultured in an incubator at 37°C with 5% CO_2_. C6/36 cells were cultured in Leibovitz-15 medium (L-15 medium) (Sigma-Aldrich) in an incubator at 28°C without additional CO_2_.

### Viruses

For this study we used DENV2 strain 16681 (GenBank accession no. NC_001474.2) and ZIKV strain PRVABC59 (GenBank accession no. KU501215.1) [27]. Wildtype and recoded viruses were rescued from their respective infectious clones by DNA-launch in BHK-21 cells (see below).

### Virus titration

Virus titration was performed in BHK-21 cells using our previously reported plaque assay [27] or fluorescent focus formation assay [41]. For plaque assay, BHK-21 cells were seeded one day before virus inoculation in a 24-well plate at a density of 5 x 10^4^ cells per well. To prepare for virus inoculation, virus stocks were serially diluted 10-fold in RPMI medium supplemented with 2% FCS and 2g/L of NaHCO3. Next, the cell culture medium was removed from the cells, and then each well was inoculated with 100 μl of the serially diluted virus stock. The cells and virus were then incubated in an incubator at 37°C with 5% CO_2_ for 1 hour. After incubation, the supernatant containing the virus was removed from the wells, and then the cells were washed twice with 1 ml of PBS per well. After washing, the cells were overlaid with RPMI medium supplemented with 2% FCS, 2g/L of NaHCO3, and 0.8% CMC. The inoculated cells were then incubated in an incubator at 37°C with 5% CO_2_ for 8 days. After incubation, the cells and plaques were fixed and stained with a solution containing 10% paraformaldehyde and 1% crystal violet.

For fluorescent focus formation assay, BHK-21 cells were seeded one day before virus inoculation in a 96-well plate at a density of 1.2 x 10^4^ cells per well. For inoculation, the cell culture medium was removed from the cells, and then each well was inoculated with 40 μl of neat virus stock. The cells and virus were then incubated in an incubator at 37°C with 5% CO_2_ for 1 hour. After incubation, the supernatant containing the virus was removed from the wells, and then the cells were washed twice with PBS. After washing, the cells were overlaid with RPMI medium supplemented with 2% FCS and 2g/L of NaHCO_3_. The inoculated cells were then incubated in an incubator at 37°C with 5% CO_2_ for 2 days. After incubation, the cells were fixed using a 4% PFA solution and their nuclei were stained with DAPI. After fixation, the cells were analysed by fluorescent microscopy using an automated Operetta High content imager platform (PerkinElmer) [41]. The fluorescent microscopy images were then analysed using the Cell Profiler software to determine the ratio of GFP positive cells to total nuclei count and this ratio was used to calculate the virus concentration in terms of focus forming units per ml (FFU/ml) [41].

### Virus culture and viral growth kinetics

The virus culture media that was used for virus culture and growth kinetics was the same as the cell culture media of the respective cell line, except that the concentration of FCS was reduced from 10% to 2%.

Viral growth kinetics was performed in BHK-21, Huh-7, HepG2, Vero, and C6/36 cells. The multiplicity of infection (MOI) for experiments was determined by the virus stock with the lowest titre. The cells were seeded one day before virus inoculation in a 24-well plate. Seeding densities per well are as follows: 6 x 10^4^ cells for BHK-21, 8 x 10^4^ cells for Huh-7, 1.8 x 10^5^ cells for HepG2, 9.0 x 10^4^ cells for Vero, and 2.5 x 10^5^ cells for C6/36. To prepare for virus inoculation, the virus stocks were diluted to the appropriate concentration in the virus culture medium of the cell line that was to be infected. Next, the cell culture medium was removed from the 24-well plates, and then each well was inoculated with 200 μl of the diluted virus stock. The cells and virus were then incubated in an incubator at 37°C with 5% CO2 for 1 hour. After incubation, the supernatant containing the virus was removed from the wells, and then the cells were washed twice with 1 ml of PBS per well. After washing, 1ml of the appropriate virus culture medium was added to each well. The inoculated human and mammalian cells were then incubated in an incubator at 37°C with 5% CO2, while the inoculated C6/36 cells were incubated in an incubator at 28°C. The virus supernatant was harvested once per day after infection until all or almost all of the cells that were infected with wildtype virus developed cytopathic effects.

### Construction of recoded infectious clones

The infectious clones described in this study were derived from our existing DENV2 and ZIKV infectious clones [27,41]. These include the previously reported EGFP reporter dengue virus 2 (DENV2-EGFP), Firefly luciferase (FLuc) translation reporter, and DENV2 subgenomic replicon [41].

The codon optimisation process can be performed using any online codon optimisation tool. For this study, the codon optimisation service and gene synthesis was performed by GenScript Biotech. The details of the regions targeted for recoding are detailed in **Table 1**. The sequence of DENV2-rcCap-NS1, the most extensively recoded clone, is also available on Genbank (accession number OP909734). The recoded DENV2 cDNA sequences were synthesised as short, slightly overlapping fragments of a few hundred base pairs in length. The recoded ZIKV cDNA sequences were synthesised as overlapping fragments of a few thousand base pairs in length. The recoded sequences were assembled using fusion PCR (Q5 Hot Start High-Fidelity 2X Master Mix, NEB). The assembled sequences were cloned into infectious clone plasmids using conventional molecular cloning techniques: DNA was digested with restriction enzymes (NEB) and ligated using T4 ligase (NEB). Infectious clone plasmids were propagated in Stbl3 *E*. *coli* competent cells (Thermo Fisher) that were cultured in LB broth supplemented with 35 μg/ml of kanamycin (GoldBio). The infectious clone plasmid sequences were verified by Sanger sequencing (performed by 1st BASE, Axil Scientific).

As reported in our previous study, the reporter DENV2-EGFP encodes a recombinant C75-EGFP-P2A-UBB-smC75 cassette, whereby the first 75 nucleotides of the capsid coding regions are duplicated to give the “C75” and “smC75” sequences [41]. The upstream C75 sequence preserves the wildtype sequence and position of two critical RNA elements, the capsid coding region hairpin element (cHP) and the 5′ cyclisation sequence (5′CS). The downstream smC75 sequence is codon optimised to abrogate the duplicated cHP and 5′CS with multiple silent mutations (essentially recoding them). The EGFP gene is cloned between these two duplicate regions. For the recoded DENV2 clones that do not encode EGFP, the first 75 nucleotides of the capsid coding region are not codon optimised to avoid targeting the critical 5′CS and cHP elements as we suspect that there would be strong selection pressure for reversion mutations if we did codon optimise them.

We note that in the design of the DENV2-rcCap-Env clone, the first 75 nucleotides of the Capsid coding region and last 75 nucleotides of the Envelope coding region are deliberately left unmodified, and only the codons that lie in between these two regions are recoded. This design is derived from prior flavivirus subgenomic replicons, which are constructed by deleting this same stretch of nucleotides lie between the first 75 nucleotides of the capsid coding region and the last 75 nucleotides of the envelope coding region [55]. These subgenomic replicons lack the expression of structural proteins but retain the ability to undergo RNA replication, indicating that the RNA elements in the deleted region are not strictly essential for RNA replication [55].

### Virus rescue from infectious clones

Virus rescue from infectious clone plasmids was performed by DNA launch in BHK-21 cells, as described in our previous study [27]. BHK-21 cells were seeded one day before transfection in a 6-well plate at a density of 2.4 x 10^5^ cells per well. Infectious clones were DNA launched by co-transfecting the infectious clone plasmid with the pTet-Off Advanced accessory plasmid (400 ng of viral plasmid for every 100 ng of accessory plasmid). The transfection was performed using jetPRIME (Polyplus transfection) according to the manufacturer’s instructions; each well was transfected with a total of 2,000 ng of DNA. Five hours after transfection, the cell culture medium was changed to virus culture medium. The transfected cells were then incubated in an incubator at 37°C with 5% CO2. The virus supernatant was harvested when the cells started to show CPE: the virus supernatant was filtered using a Sartorius syringe driven 0.22 micron PES filter and then aliquoted before being stored at -80°C. We consider this to be the passage 1 virus stock.

### Fluorescent microscopy

Live imaging of infected cells was performed using EGFP reporter DENV2 (DENV2-EGFP). The wildtype and recoded DENV2-EGFP clones were DNA-launched in BHK-21 cells as described above. The cells were imaged live using fluorescent microscopy was performed on an EVO FL digital inverted fluorescence microscope (Thermo Fisher).

### Translation reporter luciferase assay and subgenomic replicon RNA replication assay

The FLuc translation reporter construct was used to study the impact of recoding on viral protein translation efficiency. The translation reporter construct is can undergo viral protein expression, but is unable to undergo RNA replication due to a deletion of the GDD catalytic triad RNA-dependent RNA polymerase domain of the NS5 protein (**Fig 1B**) [41]. The translation reporter construct expresses firefly luciferase as a reporter of viral protein expression. The firefly luciferase assay is the same as previously described [41].

The subgenomic replicon was used to study the impact of recoding on viral RNA replication. The subgenomic replicon retains the ability to undergo viral protein expression and RNA replication but is limited to a single round of infection because it contains a partial deletion of the Capsid protein. The qRT-PCR assay that was used to measure replicon RNA replication efficiency is the same as previously described [41].

### Viral RNA extraction and fragmentation for Next generation sequencing

For each sample, viral RNA was extracted from 200 μl of viral supernatant. First, 600 μl of lysis buffer (Invitrogen) was added to each sample. The mixture was vortexed and left to incubate on ice for 10 minutes. After incubation, 800 μl of acid phenol:chloroform (Ambion: 5:1, pH 4.5) was added. The mixture was vortexed again and then centrifuged for 5 minutes at 4°C to separate the phenol-chloroform phase, the interphase, and the aqueous phase. The aqueous phase was transferred to a new tube. An equal volume of 100% isopropanol and 2 μl of Pellet Paint Co-Precipitant (Novogene) was added to the aqueous phase and the mixture was incubated at room temperature for 5 minutes and then centrifuged for 10 minutes to pellet the RNA. Next, the supernatant was removed, leaving the RNA pellet. The RNA pellet was then washed three times with 70% ethanol, followed by another three washes with 100% ethanol. The final ethanol wash was then removed and the RNA pellet was allowed to air dry. Finally, the RNA was resuspended in 30 μl of nuclease-free water.

Viral RNA fragmentation was performed using the NEBNext Magnesium RNA Fragmentation Module (NEB), according to the manufacturer’s instructions. Viral RNA was fragmented in 2 μl of NEBNext Magnesium RNA Fragmentation Buffer at 94°C for two minutes, after which the reaction was stopped by adding 2 μl of NEBNext Fragmentation Stop solution. To purify the fragmented RNA, 60 μl of 100% ethanol, 2 μl of 3M sodium acetate, and 2 μl of Pellet Paint was added to each sample. The mixture was incubated at room temperature for 5 minutes, after which the mixture was centrifuged at 4°C for 10 minutes to pellet the RNA. The supernatant was then removed and the RNA pellet was washed once with 70% ethanol and then washed one more time with 100% ethanol. The RNA pellet was allowed to air dry before being resuspended in 15 μl of nuclease-free water.

### Viral cDNA synthesis and library prep for NGS

Double stranded cDNA was synthesised from the fragmented viral RNA samples using the Maxima H Minus Double-Stranded cDNA Synthesis Kit (Thermo Fisher), according to the manufacturer’s instructions. The first strand cDNA synthesis was primed using random hexamer primers. After double stranded cDNA synthesis, residual RNA was removed by adding 10 μl of RNase I to the 100 μl double stranded cDNA mixture. The RNase reaction was incubated at room temperature for five minutes, after which the double stranded cDNA was purified using the NucleoSpin Gel and PCR Clean-up kit (Macherey-Nagel). Finally, the purified cDNA was eluted in 55 μl of Tris-HCl.

Library preparation was performed using the KAPA HyperPrep Kit (Roche Sequencing Solutions; USA) according to the SeqCap EZ HyperCap Workflow. End repair and A-tailing was performed on the samples for adapter ligation. The ligated products were cleaned up using Agencourt AMPure XP beads. The samples were then amplified using ligation-mediated polymerase chain reaction (LM-PCR), after which the samples were purified using Agencourt AMPure XP beads. Finally, Agencourt AMPure XP beads were used for size selection for fragments that were 250 bp to 450 bp in size.

The library was quantified using the Qubit dsDNA High Sensitivity Assay Kit and Qubit fluorometer (Thermo Fisher) while the library quality was verified using the Agilent 2100 Bioanalyzer.

### Next generation sequencing

The samples were sequenced by GENEWIZ using NovaSeq 6000 (Illumina). Reads were filtered using Genome Detective (v1.132) and alignment analysis was conducted using Geneious Prime (v2021.0.3) (Biomatters).

### RNA pseudoknot structure prediction and visualisation

RNA pseudoknot structures were modelled using pKiss [44], using the thermodynamic parameters published by Andronescu *et al* [45]. The predicted pseudoknot structures were visualised using PseudoViewer [46].

### SHAPE-MaP structure probing of DENV2 viruses in BHK-2 cells

BHK-21 cells were infected with DENV2 (WT and mutant) at a multiplicity of infection (MOI) = 0.01 for 1 h at 37°C. Following 1 h infection, virus inoculum was removed and replaced with DMEM-5% FBS. Flasks were incubated for 48 h at 37°C, 5% CO2. At 48 hours post infection, cells were washed once with PBS and trypsin was added to detach the cells from the flask. The cells were collected and centrifuged at 300 × g for 5min. The pellet was resuspended in PBS and the cells were then separated into three reactions: (1) added 1:20 volume of 1M NAI (03–310, Merck) and incubated for 15 min at 37°C for structure probing; (2) added 1:20 volume of dimethyl sulfoxide (DMSO) and incubated for 15 min at 37°C, as negative control; and (3) set aside a third portion of the infected cells without any treatment, for the denaturing control in the downstream library preparation process. One set of uninfected BHK-21 cells were treated as negative control. The total RNA was extracted from the cells using Qiagen RNeasy Mini Kit according to the manufacturer’s instructions. We then performed library preparation following the SHAPE-MaP protocol to generate cDNA libraries compatible for Illumina sequencing [47].

### In silico RNA secondary structure prediction and analysis

SHAPE data was obtained using Shapemapper 2.15 independently for 2 technical replicates of the Wildtype and recoded strains respectively [56]. Minimum read depth was set to 1000. Reactivity data at this depth was obtained for 98.8% of Wildtype and 99.2% of recoded RNA positions. Wildtype and recoded genomic RNA sequences were aligned using MAFFT v7.481, yielding a gapless alignment [57]. Differences in SHAPE were assessed using ΔSHAPE [58]. Comparison of SHAPE data confirmed that significant structural changes are largely confined to the recoded region spanning bases 1–2421 in both variants. Therefore, structure modelling was confined to this section. We used the Superfold pipeline with RNAstructure v6.3 as a backend for structure prediction with default parameters incorporating SHAPE data as a constraint [48]. Subsequent analysis shows good agreement between SHAPE data and the resulting RNA structure models, indicating that the models are plausible in light of the experimental evidence. Structures were visualized using VARNA 3.93 and a custom script to map SHAPE reactivity data onto the resulting figures [49].

### Neurovirulence studies in suckling mice

The *in vivo* attenuation of DENV2 was characterised in a suckling mouse model of neurovirulence that we have described in a previous study [27]. Newborn outbred white ICR mice from InVivos, Singapore, were inoculated with virus via intracranial injection within 24 hours of birth.

### Mosquito challenge studies

Mosquito challenge studies was performed by feeding an infectious blood meal to *Aedes albopictus* mosquitoes [30]. The *Aedes albopictus* (NEA-EHI strain) colony used for this study is a local Singapore strain that was obtained from the Environmental Health Institute, Singapore. The mosquito colony was maintained in the insectary of Temasek Life Sciences Laboratory using the same conditions that we have previously reported [59]. The blood meal was prepared from rabbit blood that was freshly drawn on the day of oral infection. The blood was centrifuged at 2,500 rpm and 4°C for 10 min to separate there blood cells from the serum. The serum was then heat-inactivated at 55°C for 1 hour. The blood cells were washed three times with PBS. The heat-inactivated serum and washed blood cells were then mixed together and supplemented with 1mM of ATP. The treated blood was then mixed with diluted virus stock at a 1:1 ratio, to obtain an infectious titre of 2.5 x 10^7 PFU/ml.

Oral infection was performed using female *Aedes albopictus* mosquitoes at day 5 after emergence. The mosquitoes were sugar starved overnight prior to the oral infection. The mosquitoes were fed the infectious blood meal using the Hemotek system (PS5, Hemotek Ltd England). After oral infection, partially engorged or unfed mosquitoes were removed from the cage. The remaining engorged females were kept for 11 days at 28°C with 80% humiditiy and a photoperiod of 12:12 hours (light:dark) with 10% sucrose solution provided ad libitum. After an 11 day incubation period, the mosquitoes were collected to determine their infection status and viral load. Individual mosquitoes were homogenised in 100 μl of PBS after which their infection status and viral load was determined using plaque assay.

### Nucleotide sequence alignment

Nucleotide sequence alignment was performed using Clustal Omega, using the Clustal Omega web service [60,61].

### Statistical analysis

Statistical analysis and graph plotting was performed using GraphPad Prism 9. Viral growth kinetic titres, firefly luciferase assay, qRT-PCR assay, and viral plaque sizes were compared using one-way ANOVA, with Tukey’s multiple comparisons post hoc test. Figures for the Viral growth kinetic titres, firefly luciferase assay, and qRT-PCR assay indicate the mean and standard deviation of three technical replicates and are representative of at least two biological replicates. Plaque sizes were measured in ImageJ using the ViralPlaque Fiji macro [62]. Figures for plaque sizes indicate the mean and standard deviation of at least thirty plaques and are representative of at least two biological replicates. One-way Anova with Bonferroni correction was used when performing multiple comparisons for mouse survival. Non-parametric unpaired T-test was used for mosquito challenge.

## Supporting information

S1 Fig Characterisation of DENV2 genome recoding using DENV2-EGFP. DENV2-EGFP is a dengue reporter virus that expresses EGFP. **(a)** Genomic maps showing regions of the DENV2-EGFP genome recoded with silent mutations. rcE2-90 and rcE2 clones: partial recoding of 3′ segment of Env protein coding region. rcNS1 clone: recoding of NS1 protein coding region, with partial overlap into Env and NS2A coding regions. **(b) & (c)** Fluorescent microscopy analysis of BHK-21 cells infected with recoded DENV2-EGFP (10x magnification) at day 5, 6 post infection respectively. Green fluorescent signal indicates DENV2-EGFP infected cells. Mock: mock infected control cells. WT: cells infected with wildtype (non-recoded) DENV2-EGFP.
(PDF)

S2 Fig Characterisation of putative DENV2 envelop stem RNA element (ESRE). **(a)** Genomic maps showing regions of the DENV2-EGFP genome recoded with silent mutations. The recoding targets a segment near the 3′ end of the Env protein coding region. The number at the end of each clone indicates the number of codons targeted for recoding. **(b)** Fluorescent microscopy analysis of BHK-21 cells infected with recoded DENV2-GFP at 4 days post infection (10x magnification). **(c)** Predicted RNA secondary structure of putative ESRE in wildtype DENV2-16681. Black arrows indicate the nucleotides that are mutated in the rcE2-50 recoded clone.
(PDF)

S3 Fig Comparison of recoded and wildtype sequences from DENV2 and ZIKV clones. Multiple sequence alignments were used to compare the protein coding regions of wildtype and recoded viral genomic sequences. Clustal Omega was used to create the multiple sequence alignment. **(a)** and **(b)** Multiple sequence alignment of part of the prM and Env coding sequences of wildtype DENV2 and the recoded DENV2-rcCap-prM, DENV2-rcCap-Env, and DENV2-rcCap-NS1 clones. The DENV2-rcCap-prM and DENV2-rcCap-Env clones are derived from the same master sequence, which is the DENV2-rcCap-NS1 clone. Therefore, the regions that are recoded will share the same recoding mutations. In other words, the DENV2-rcCap-prM and DENV2-rcCap-Env clones act to narrow down the region of recoding seen in the DENV2-rcCap-NS1 clone. **(a)** Nucleotides 601 to 780 of the DENV2 polyprotein coding region, corresponding to codons 87 to 146 of the prM coding region. **(b)** Nucleotides 1201 to 1380 of the DENV2 polyprotein coding region, corresponding to codons 121 to 180 of the Env coding region. **(c)** and **(d)** Multiple sequence alignment of part of the prM and NS4B coding sequences of wildtype ZIKV and the recoded ZIKV-rcprM-NS3, ZIKV-rcprM-NS5, ZIKV-rcCap-NS3, and ZIKV-Cap-NS5 clones. The ZIKV-rcprM-NS3, ZIKV-rcprM-NS5, and ZIKV-rcCap-NS3 clones are derived from the same master sequence, which is the ZIKV-rcCap-NS5 clone. Therefore, the regions that are recoded will share the same recoding mutations. **(c)** Nucleotides 601 to 780 of the ZIKV polyprotein coding region, corresponding to codons 79 to 138 of the prM coding region. **(d)** Nucleotides 6901 to 7080 of the ZIKV polyprotein coding region, corresponding to codons 32 to 91 of the NS4B coding region.
(PDF)

S4 Fig Genomic maps of rescue mutants for wildtype DENV2 and DENV2-rcCap-Env. Approximate location of recapitulatory rescue mutations are shown above the genome. Approximate region of recoding is shown below the genome.
(PDF)

S5 Fig Plaque size phenotype of recoded and rescue mutants of DENV2. **(a)** Plaques formed by wildtype DENV2, wildtype DENV2 with Env-M196V cell line adaptation mutation (WT+rsEnv), recoded DENV2 (rcCap-Env), and rescue mutants of DENV2-rcCap-Env (+rsCE, +rsCap, and +rsEnv). **(b)** Plaque sizes were measured in ImageJ using ViralPlaque Fiji macro.
(PDF)

S6 Fig Predicted RNA secondary structures in the 5′UTR and structural protein coding region of wildtype DENV2-16681, corresponding to nucleotides 1 to 2421 of the DENV2 genome. *In silico* modelling of DENV2 genomic RNA secondary structures was performed using the Superfold pipeline with RNAstructure v6.3 as the backend, with the results of our SHAPE analysis incorporated as a constraint. RNA structures were then visualized using VARNA 3.93 and a custom script to map SHAPE reactivity data onto the resulting figure. Red colour indicates increased reactivity.
(PDF)

S7 Fig Predicted RNA secondary structures in the 5′UTR and structural protein coding region of DENV2-rcCap-Env+rsCE recoded rescue clone. *In silico* modelling of DENV2 genomic RNA secondary structures was performed using the Superfold pipeline with RNAstructure v6.3 as the backend, with the results of our SHAPE analysis incorporated as a constraint. RNA structures were then visualized using VARNA 3.93 and a custom script to map SHAPE reactivity data onto the resulting figure. Red colour indicates increased reactivity.
(PDF)

S1 Table Statistical analysis of growth kinetics of the recoded viruses relative to wildtype virus in cell culture shown in Fig 2B. Statistical analysis of mean viral titre (n = 3) of the recoded viruses relative to wildtype for each day post infection was performed using one-way ANOVA, and post-hoc analysis was performed using Tukey HSD. Statistical significance is abbreviated as: n.s, not significant; *, P<0.05; **, P<0.01; ***, P<0.001. ND indicates virus titre was below the limit of detection of 10 PFU/ml. SD: standard deviation.
(DOCX)

S2 Table Statistical analysis of growth kinetics of the recoded viruses relative to wildtype virus in cell culture shown in Fig 4C. Statistical analysis of mean viral titre (n = 3) of the recoded viruses relative to wildtype for each day post infection was performed using one-way ANOVA, and post-hoc analysis was performed using Tukey HSD. Statistical significance is abbreviated as: n.s, not significant; *, P<0.05; **, P<0.01; ***, P<0.001. ND indicates virus titre was below the limit of detection of 10 PFU/ml. SD: standard deviation.
(DOCX)

S3 Table Statistical analysis of growth kinetics of the recoded viruses relative to wildtype virus in cell culture shown in Fig 5B. Statistical analysis of mean viral titre (n = 3) of the recoded viruses relative to wildtype for each day post infection was performed using one-way ANOVA, and post-hoc analysis was performed using Tukey HSD. Statistical significance is abbreviated as: n.s, not significant; *, P<0.05; **, P<0.01; ***, P<0.001. ND indicates virus titre was below the limit of detection of 10 PFU/ml. SD: standard deviation.
(DOCX)
