# Supplementary figures and images for: Flavivirus genome recoding by codon optimisation confers genetically stable in vivo attenuation in both mice and mosquitoes
Source: PLoS Pathog. 2023 Oct 26;19(10):e1011753. doi: 10.1371/journal.ppat.1011753 (PMC10629665; doi:10.1371/journal.ppat.1011753)

Fig S1

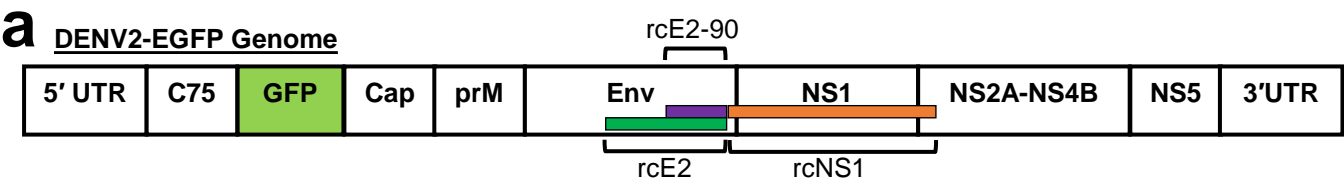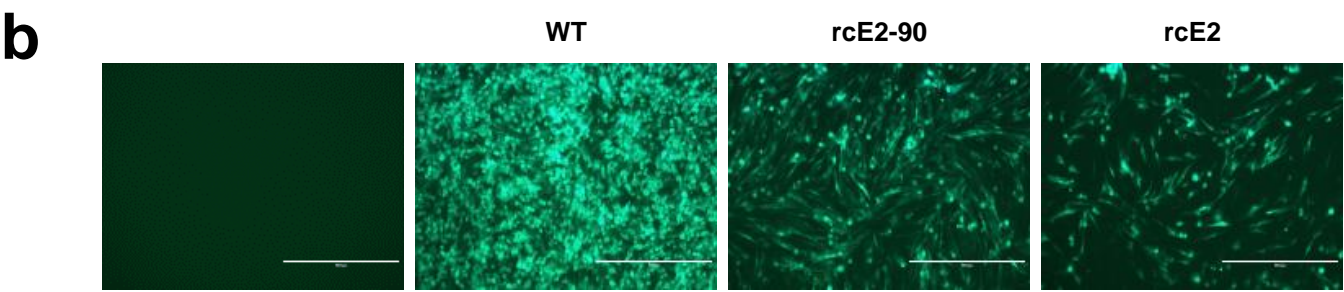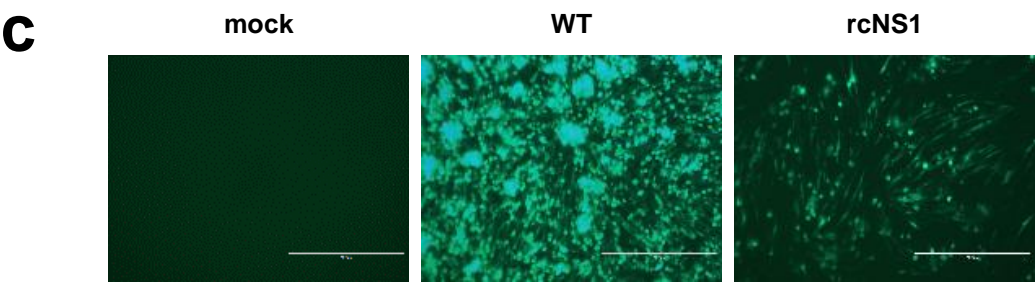

Supplement: S1 Fig — DENV2-EGFP is a dengue reporter virus that expresses EGFP. (a) Genomic maps showing regions of the DENV2-EGFP genome recoded with silent mutations. rcE2-90 and rcE2 clones: partial recoding of 3′ segment of Env protein coding region. rcNS1 clone: recoding of NS1 protein coding region, with partial overlap into Env and NS2A coding regions. (b) & (c) Fluorescent microscopy analysis of BHK-21 cells infected with recoded DENV2-EGFP (10x magnification) at day 5, 6 post infection respectively. Green fluorescent signal indicates DENV2-EGFP infected cells. Mock: mock infected control cells. WT: cells infected with wildtype (non-recoded) DENV2-EGFP. (PDF) [file ppat.1011753.s001.pdf]

**a**

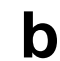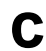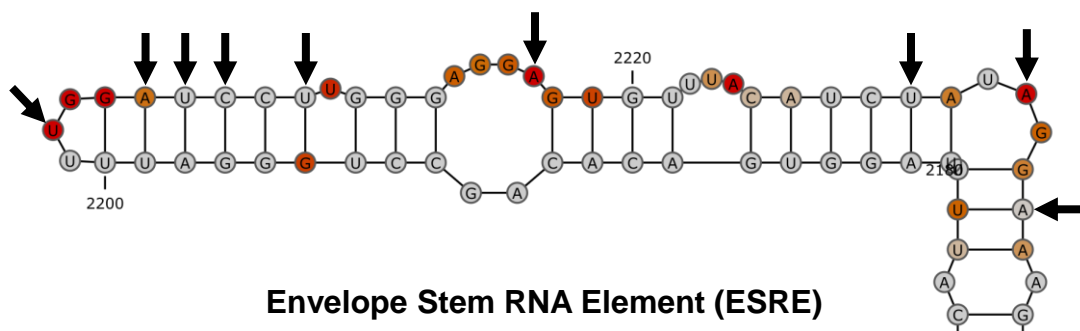

Supplement: S2 Fig — (a) Genomic maps showing regions of the DENV2-EGFP genome recoded with silent mutations. The recoding targets a segment near the 3′ end of the Env protein coding region. The number at the end of each clone indicates the number of codons targeted for recoding. (b) Fluorescent microscopy analysis of BHK-21 cells infected with recoded DENV2-GFP at 4 days post infection (10x magnification). (c) Predicted RNA secondary structure of putative ESRE in wildtype DENV2-16681. Black arrows indicate the nucleotides that are mutated in the rcE2-50 recoded clone. (PDF) [file ppat.1011753.s002.pdf]

Fig S5

a

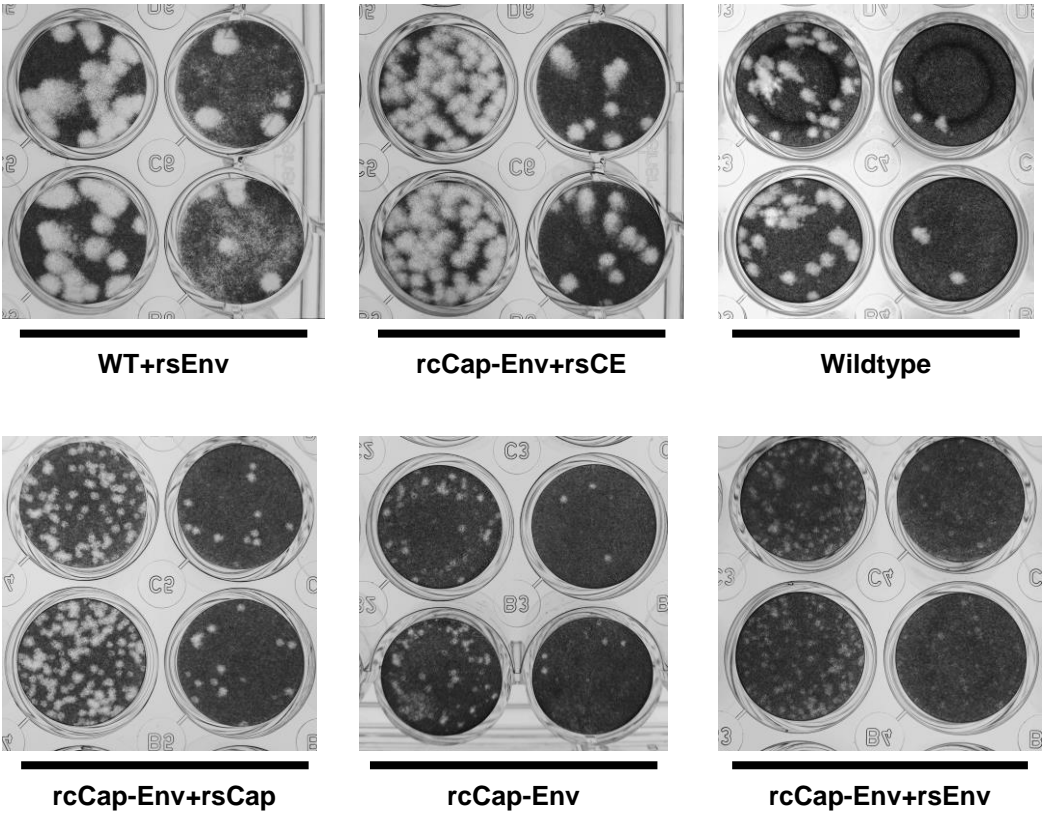

b

Rescue Clone Plaque Size

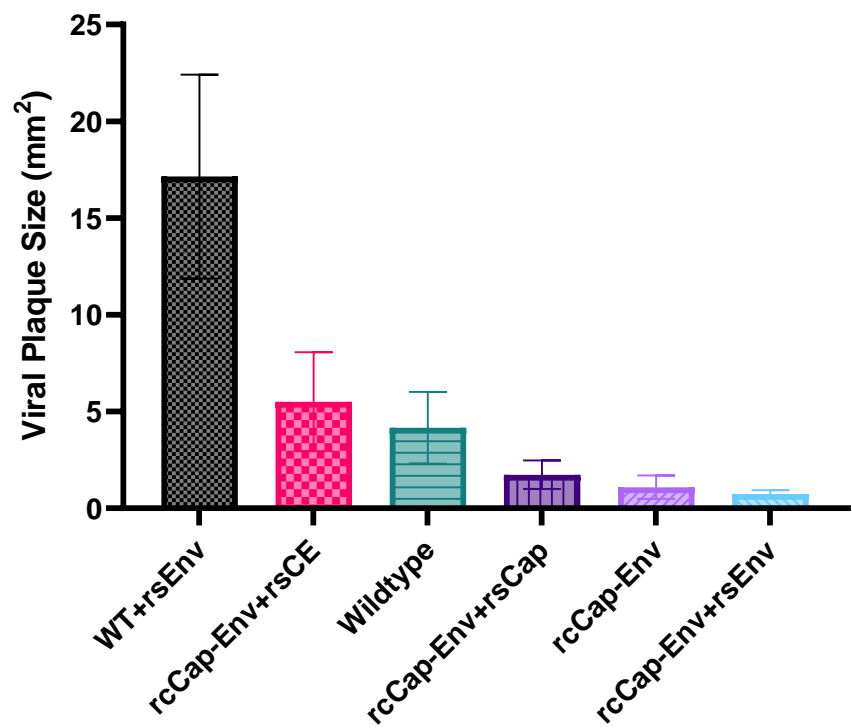

Supplement: S5 Fig — (a) Plaques formed by wildtype DENV2, wildtype DENV2 with Env-M196V cell line adaptation mutation (WT+rsEnv), recoded DENV2 (rcCap-Env), and rescue mutants of DENV2-rcCap-Env (+rsCE, +rsCap, and +rsEnv). (b) Plaque sizes were measured in ImageJ using ViralPlaque Fiji macro. (PDF) [file ppat.1011753.s005.pdf]

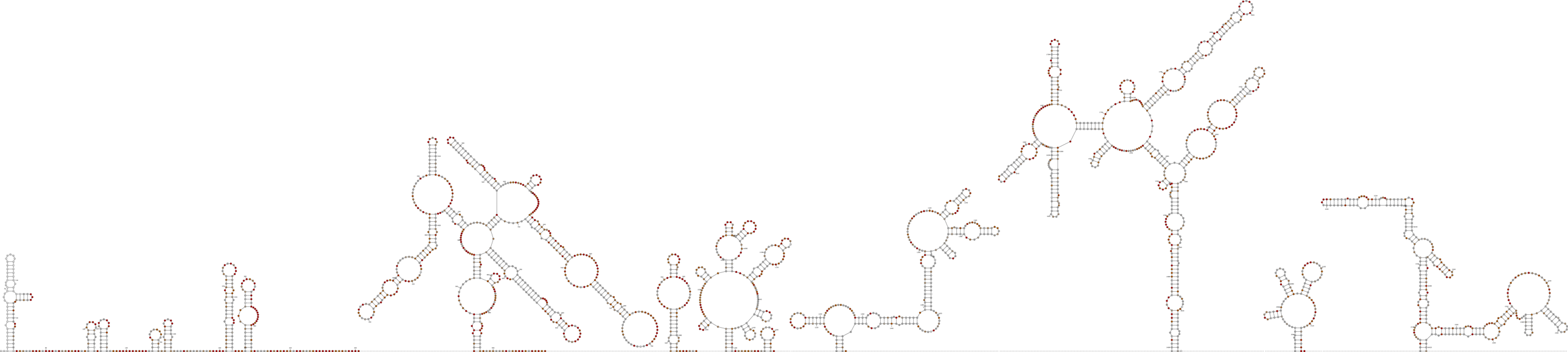

Supplement: S6 Fig — In silico modelling of DENV2 genomic RNA secondary structures was performed using the Superfold pipeline with RNAstructure v6.3 as the backend, with the results of our SHAPE analysis incorporated as a constraint. RNA structures were then visualized using VARNA 3.93 and a custom script to map SHAPE reactivity data onto the resulting figure. Red colour indicates increased reactivity. (PDF) [file ppat.1011753.s006.pdf]

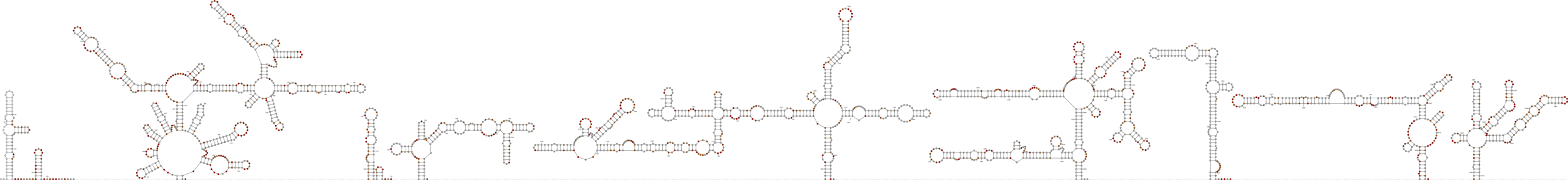

Supplement: S7 Fig — In silico modelling of DENV2 genomic RNA secondary structures was performed using the Superfold pipeline with RNAstructure v6.3 as the backend, with the results of our SHAPE analysis incorporated as a constraint. RNA structures were then visualized using VARNA 3.93 and a custom script to map SHAPE reactivity data onto the resulting figure. Red colour indicates increased reactivity. (PDF) [file ppat.1011753.s007.pdf]
