# Supplementary material for: Flavivirus genome recoding by codon optimisation confers genetically stable in vivo attenuation in both mice and mosquitoes
Source: PLoS Pathog. 2023 Oct 26;19(10):e1011753. doi: 10.1371/journal.ppat.1011753 (PMC10629665; doi:10.1371/journal.ppat.1011753)
Supplement: S3 Fig — Multiple sequence alignments were used to compare the protein coding regions of wildtype and recoded viral genomic sequences. Clustal Omega was used to create the multiple sequence alignment. (a) and (b) Multiple sequence alignment of part of the prM and Env coding sequences of wildtype DENV2 and the recoded DENV2-rcCap-prM, DENV2-rcCap-Env, and DENV2-rcCap-NS1 clones. The DENV2-rcCap-prM and DENV2-rcCap-Env clones are derived from the same master sequence, which is the DENV2-rcCap-NS1 clone. Therefore, the regions that are recoded will share the same recoding mutations. In other words, the DENV2-rcCap-prM and DENV2-rcCap-Env clones act to narrow down the region of recoding seen in the DENV2-rcCap-NS1 clone. (a) Nucleotides 601 to 780 of the DENV2 polyprotein coding region, corresponding to codons 87 to 146 of the prM coding region. (b) Nucleotides 1201 to 1380 of the DENV2 polyprotein coding region, corresponding to codons 121 to 180 of the Env coding region. (c) and (d) Multiple sequence alignment of part of the prM and NS4B coding sequences of wildtype ZIKV and the recoded ZIKV-rcprM-NS3, ZIKV-rcprM-NS5, ZIKV-rcCap-NS3, and ZIKV-Cap-NS5 clones. The ZIKV-rcprM-NS3, ZIKV-rcprM-NS5, and ZIKV-rcCap-NS3 clones are derived from the same master sequence, which is the ZIKV-rcCap-NS5 clone. Therefore, the regions that are recoded will share the same recoding mutations. (c) Nucleotides 601 to 780 of the ZIKV polyprotein coding region, corresponding to codons 79 to 138 of the prM coding region. (d) Nucleotides 6901 to 7080 of the ZIKV polyprotein coding region, corresponding to codons 32 to 91 of the NS4B coding region. (PDF) [file ppat.1011753.s003.pdf]

# Fig S3

**a** DENV2 ORF Nucleotides 601 to 780

```
WT DENV2     AGAAGAGAAAAAAGATCAGTGGCACTCGTTCCACATGTGGGAATGGGACTGGAGACACGA  660
rcCap-prM    CGGAGAGAGAAGCGGAGCGTGGCCCTGGTGCCCCACGTGGGAATGGGCCTGGAGACCAGA  660
rcCap-Env    CGGAGAGAGAAGCGGAGCGTGGCCCTGGTGCCCCACGTGGGAATGGGCCTGGAGACCAGA  660
rcCap-NS1    CGGAGAGAGAAGCGGAGCGTGGCCCTGGTGCCCCACGTGGGAATGGGCCTGGAGACCAGA  660
              * ***** **  *    ***** ** ** ** *********** ********  **

WT DENV2     ACTGAAACATGGATGTCATCAGAAGGGGCCTGGAAACATGTCCAGAGAATTGAAACTTGG  720
rcCap-prM    ACAGAGACCTGGATGAGCTCCGAGGGAGCATGGAAGCACGTGCAGCGCATCGAGACATGG  720
rcCap-Env    ACAGAGACCTGGATGAGCTCCGAGGGAGCATGGAAGCACGTGCAGCGCATCGAGACATGG  720
rcCap-NS1    ACAGAGACCTGGATGAGCTCCGAGGGAGCATGGAAGCACGTGCAGCGCATCGAGACATGG  720
             ** ** ** ******   ** ** ** ** ***** ** ** *** * ** ** ** ***

WT DENV2     ATCTTGAGACATCCAGGCTTCACCATGATGGCAGCAATCCTGGCATACACCATAGGAACG  780
rcCap-prM    ATTCTGAGGCACCCCGGCTTCACCATGATGGCAGCCATCCTGGCCTACACAATCGGCACC  780
rcCap-Env    ATTCTGAGGCACCCCGGCTTCACCATGATGGCAGCCATCCTGGCCTACACAATCGGCACC  780
rcCap-NS1    ATTCTGAGGCACCCCGGCTTCACCATGATGGCAGCCATCCTGGCCTACACAATCGGCACC  780
             **  **** ** ** ******************** ******** ***** ** ** **
```

**b** DENV2 ORF Nucleotides 1201 to 1380

```
WT DENV2     TGCAAAAAGAACATGGAAGGAAAAGTTGTGCAACCAGAAAACTTGGAATACACCATTGTG  1260
rcCap-prM    TGCAAAAAGAACATGGAAGGAAAAGTTGTGCAACCAGAAAACTTGGAATACACCATTGTG  1260
rcCap-Env    TGTAAGAAGAATATGGAGGGCAAGGTGGTGCAGCCTGAGAACCTGGAGTACACCATCGTG  1260
rcCap-NS1    TGTAAGAAGAATATGGAGGGCAAGGTGGTGCAGCCTGAGAACCTGGAGTACACCATCGTG  1260
             ** ** ***** ***** ** ** ** ***** ** ** *** **** ******** ***

WT DENV2     ATAACACCTCACTCAGGGGAAGAGCATGCAGTCGGAAATGACACAGGAAAACATGGCAAG  1320
rcCap-prM    ATAACACCTCACTCAGGGGAAGAGCATGCAGTCGGAAATGACACAGGAAAACATGGCAAG  1320
rcCap-Env    ATCACACCACACTCCGGAGAGGAGCACGCAGTGGGAAACGATACCGGCAAGCACGGCAAG  1320
rcCap-NS1    ATCACACCACACTCCGGAGAGGAGCACGCAGTGGGAAACGATACCGGCAAGCACGGCAAG  1320
             ** ***** ***** ** ** ***** ***** ***** ** ** ** ** ** ******

WT DENV2     GAAATCAAAATAACACCACAGAGTTCCATCACAGAAGCAGAATTGACAGGTTATGGCACT  1380
rcCap-prM    GAAATCAAAATAACACCACAGAGTTCCATCACAGAAGCAGAATTGACAGGTTATGGCACT  1380
rcCap-Env    GAGATCAAGATCACACCACAGTCTAGCATCACCGAGGCCGAGCTGACAGGCTATGGCACA  1380
rcCap-NS1    GAGATCAAGATCACACCACAGTCTAGCATCACCGAGGCCGAGCTGACAGGCTATGGCACA  1380
             ** ***** ** *********  *  ****** ** ** **  ******* ********
```

# Fig S3

**c** ZIKV ORF Nucleotides 601 to 780

```
WT ZIKV      GGAACCTGCCATCACAAAAAAGGTGAAGCACGGAGATCTAGAAGAGCTGTGACGCTCCCC  660
rcprm-NS3    GGCACATGTCACCACAAGAAGGGAGAGGCACGGAGATCTAGGCGCGCAGTGACCCTGCCA  660
rcprm-NS5    GGCACATGTCACCACAAGAAGGGAGAGGCACGGAGATCTAGGCGCGCAGTGACCCTGCCA  660
rcCap-NS3    GGCACATGTCACCACAAGAAGGGAGAGGCACGGAGATCTAGGCGCGCAGTGACCCTGCCA  660
rcCap-NS5    GGCACATGTCACCACAAGAAGGGAGAGGCACGGAGATCTAGGCGCGCAGTGACCCTGCCA  660
             ** ** ** ** ***** ** ** ** **************  * ** ***** ** ** 

WT ZIKV      TCCCATTCCACCAGGAAGCTGCAAACGCGGTCGCAAACCTGGTTGGAATCAAGAGAATAC  720
rcprM-NS3    AGCCACTCCACAAGGAAGCTGCAGACCCGCTCTCAGACATGGCTGGAGAGCCGGGAGTAT  720
rcprM-NS5    AGCCACTCCACAAGGAAGCTGCAGACCCGCTCTCAGACATGGCTGGAGAGCCGGGAGTAT  720
rcCap-NS3    AGCCACTCCACAAGGAAGCTGCAGACCCGCTCTCAGACATGGCTGGAGAGCCGGGAGTAT  720
rcCap-NS5    AGCCACTCCACAAGGAAGCTGCAGACCCGCTCTCAGACATGGCTGGAGAGCCGGGAGTAT  720
               *** ***** *********** ** ** ** ** ** *** ****     * ** **  

WT ZIKV      ACAAAGCACTTGATTAGAGTCGAAAATTGGATATTCAGGAACCCTGGCTTCGCGTTAGCA  780
rcprM-NS3    ACCAAGCACCTGATCCGGGTGGAGAATTGGATCTTTAGAAACCCAGGCTTCGCACTGGCT  780
rcprM-NS5    ACCAAGCACCTGATCCGGGTGGAGAATTGGATCTTTAGAAACCCAGGCTTCGCACTGGCT  780
rcCap-NS3    ACCAAGCACCTGATCCGGGTGGAGAATTGGATCTTTAGAAACCCAGGCTTCGCACTGGCT  780
rcCap-NS5    ACCAAGCACCTGATCCGGGTGGAGAATTGGATCTTTAGAAACCCAGGCTTCGCACTGGCT  780
             ** ****** ****  * ** ** ******** ** ** ***** ********  * **  
```

**d** ZIKV ORF Nucleotides 6900 to 7080

```
WT ZIKV      ATTGACCTGCGGCCAGCCTCAGCTTGGGCCATCTATGCTGCCTTGACAACTTTCATTACC  6960
rcprM-NS3    ATTGACCTGCGGCCAGCCTCAGCTTGGGCCATCTATGCTGCCTTGACAACTTTCATTACC  6960
rcprM-NS5    ATCGATCTGAGGCCAGCATCCGCCTGGGCAATCTACGCCGCCCTGACAACCTTCATCACA  6960
rcCap-NS3    ATTGACCTGCGGCCAGCCTCAGCTTGGGCCATCTATGCTGCCTTGACAACTTTCATTACC  6960
rcCap-NS5    ATCGATCTGAGGCCAGCATCCGCCTGGGCAATCTACGCCGCCCTGACAACCTTCATCACA  6960
             ** ** *** ******* ** ** ***** ***** ** *** ******* ***** ** 

WT ZIKV      CCAGCCGTCCAACATGCAGTGACCACCTCATACAACAACTACTCCTTAATGGCGATGGCC  7020
rcprM-NS3    CCAGCCGTCCAACATGCAGTGACCACCTCATACAACAACTACTCCTTAATGGCGATGGCC  7020
rcprM-NS5    CCAGCAGTGCAGCACGCAGTGACAACCAGCTACAACAATTATTCCCTGATGGCTATGGCC  7020
rcCap-NS3    CCAGCCGTCCAACATGCAGTGACCACCTCATACAACAACTACTCCTTAATGGCGATGGCC  7020
rcCap-NS5    CCAGCAGTGCAGCACGCAGTGACAACCAGCTACAACAATTATTCCCTGATGGCTATGGCC  7020
             ***** ** ** ** ******** ***   ******** ** *** * ***** ******

WT ZIKV      ACGCAAGCTGGAGTGTTGTTTGGCATGGGCAAAGGGATGCCATTCTACGCATGGGACTTT  7080
rcprM-NS3    ACGCAAGCTGGAGTGTTGTTTGGCATGGGCAAAGGGATGCCATTCTACGCATGGGACTTT  7080
rcprM-NS5    ACCCAGGCAGGCGTGCTGTTCGGAATGGGCAAGGGCATGCCTTTTTACGCCTGGGACTTC  7080
rcCap-NS3    ACGCAAGCTGGAGTGTTGTTTGGCATGGGCAAAGGGATGCCATTCTACGCATGGGACTTT  7080
rcCap-NS5    ACCCAGGCAGGCGTGCTGTTCGGAATGGGCAAGGGCATGCCTTTTTACGCCTGGGACTTC  7080
             ** ** ** ** *** **** ** ******** ** ***** ** ***** ******** 
```
