# Supplementary material for: Flavivirus genome recoding by codon optimisation confers genetically stable in vivo attenuation in both mice and mosquitoes
Source: PLoS Pathog. 2023 Oct 26;19(10):e1011753. doi: 10.1371/journal.ppat.1011753 (PMC10629665; doi:10.1371/journal.ppat.1011753)
Supplement: S4 Fig — Approximate location of recapitulatory rescue mutations are shown above the genome. Approximate region of recoding is shown below the genome. (PDF) [file ppat.1011753.s004.pdf]

Fig S4

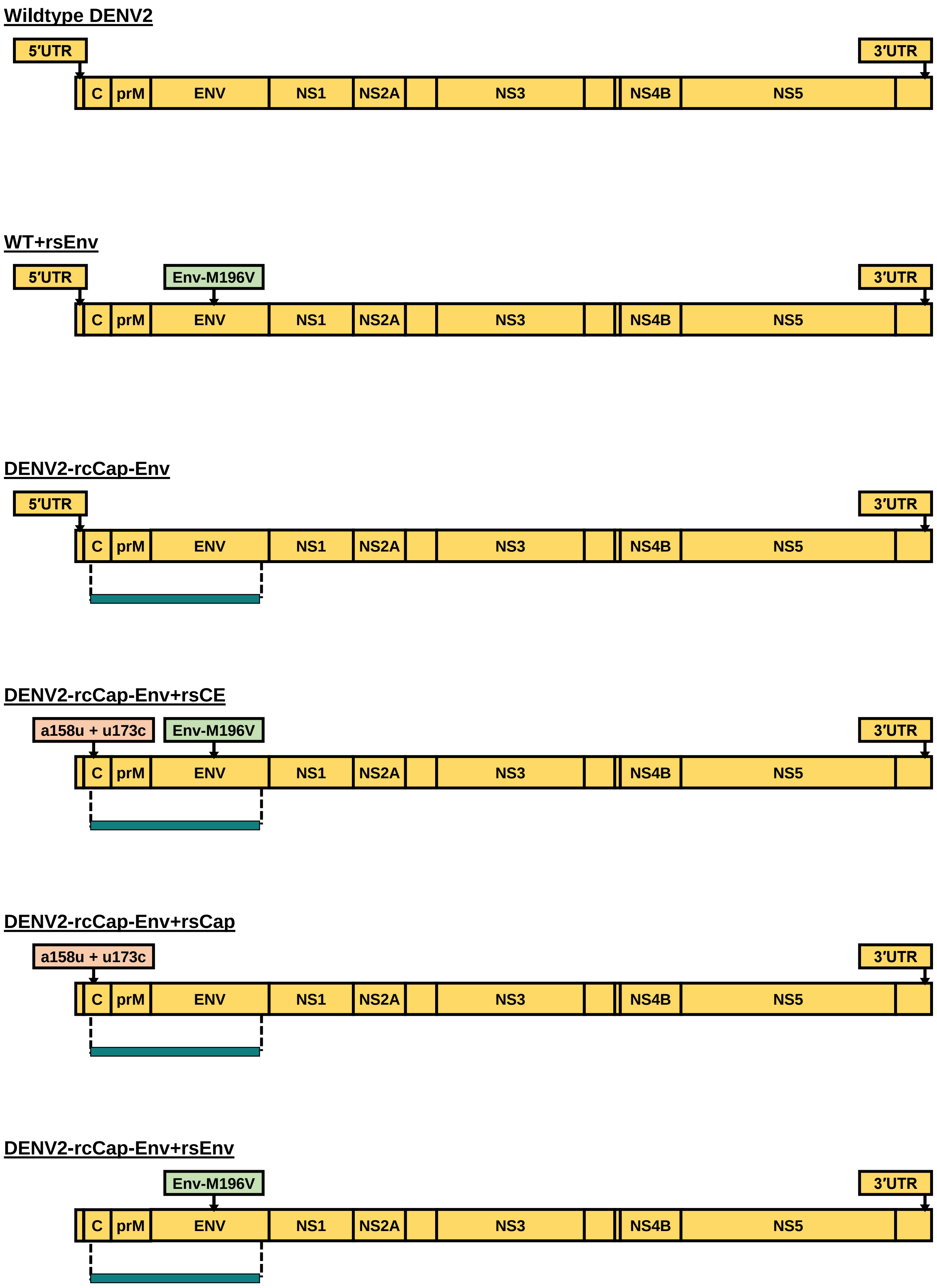
Wildtype DENV2
5′UTR
3′UTR
C
prM
ENV
NS1
NS2A
NS3
NS4B
NS5
WT+rsEnv
5′UTR
Env-M196V
3′UTR
C
prM
ENV
NS1
NS2A
NS3
NS4B
NS5
DENV2-rcCap-Env
5′UTR
3′UTR
C
prM
ENV
NS1
NS2A
NS3
NS4B
NS5
DENV2-rcCap-Env+rsCE
a158u + u173c
Env-M196V
3′UTR
C
prM
ENV
NS1
NS2A
NS3
NS4B
NS5
DENV2-rcCap-Env+rsCap
a158u + u173c
3′UTR
C
prM
ENV
NS1
NS2A
NS3
NS4B
NS5
DENV2-rcCap-Env+rsEnv
Env-M196V
3′UTR
C
prM
ENV
NS1
NS2A
NS3
NS4B
NS5
