# Supplementary material for: Flavivirus genome recoding by codon optimisation confers genetically stable in vivo attenuation in both mice and mosquitoes
Source: PLoS Pathog. 2023 Oct 26;19(10):e1011753. doi: 10.1371/journal.ppat.1011753 (PMC10629665; doi:10.1371/journal.ppat.1011753)
Supplement: S1 Table — Statistical analysis of mean viral titre (n = 3) of the recoded viruses relative to wildtype for each day post infection was performed using one-way ANOVA, and post-hoc analysis was performed using Tukey HSD. Statistical significance is abbreviated as: n.s, not significant; *, P<0.05; **, P<0.01; ***, P<0.001. ND indicates virus titre was below the limit of detection of 10 PFU/ml. SD: standard deviation. (DOCX) [file ppat.1011753.s008.docx]

BHK-21 cells, Mean viral titre (PFU/ml)

| DENV2 clone | Days post infection | | | | | | | | | | | |
| --- | --- | --- | --- | --- | --- | --- | --- | --- | --- | --- | --- | --- |
|  | 1 | | 2 | | 3 | | 4 | | 5 | | 6 | |
|  | Mean | SD | Mean | SD | Mean | SD | Mean | SD | Mean | SD | Mean | SD |
| Wildtype | 4.53E+02 | 4.73E+01 | 9.00E+04 | 4.00E+04 | 2.80E+06 | 2.00E+05 | 9.67E+05 | 1.15E+05 | 4.23E+05 | 3.06E+04 | 9.00E+04 | 4.00E+04 |
| rcCap-prM | ND | ND | 1.00E+04 | 3.61E+03 | 1.37E+05 | 1.53E+04 | 8.67E+04 | 1.15E+04 | 9.00E+05 | 1.00E+05 | 1.00E+04 | 3.61E+03 |
| rcCap-Env | ND | ND | 3.53E+03 | 1.31E+03 | 2.40E+04 | 7.94E+03 | 1.87E+04 | 4.73E+03 | 6.67E+05 | 2.52E+05 | 3.53E+03 | 1.31E+03 |
| rcCap-NS1 | ND | ND | 3.50E+03 | 2.65E+02 | 2.50E+04 | 6.56E+03 | 9.33E+04 | 5.77E+03 | 1.50E+06 | 1.73E+05 | 3.50E+03 | 2.65E+02 |

BHK-21 cells, Statistical analysis of mean viral titre relative to wildtype virus control

| DENV2 clone | Days post infection | | | | | |
| --- | --- | --- | --- | --- | --- | --- |
|  | 1 | 2 | 3 | 4 | 5 | 6 |
| rcCap-prM | *** | *** | *** | *** | ** | n.s. |
| rcCap-Env | *** | *** | *** | *** | n.s. | ** |
| rcCap-NS1 | *** | *** | *** | *** | *** | *** |

Huh-7 cells, Mean viral titre (PFU/ml)

| DENV2 clone | Days post infection | | | | | | | | | | | |
| --- | --- | --- | --- | --- | --- | --- | --- | --- | --- | --- | --- | --- |
|  | 1 | | 2 | | 3 | | 4 | | 5 | | 6 | |
|  | Mean | SD | Mean | SD | Mean | SD | Mean | SD | Mean | SD | Mean | SD |
| Wildtype | 5.07E+02 | 2.52E+01 | 2.67E+05 | 8.14E+04 | 9.33E+05 | 3.21E+05 | 3.53E+05 | 3.79E+04 | 2.40E+04 | 8.89E+03 | 1.07E+04 | 2.52E+03 |
| rcCap-prM | 1.67E+01 | 2.08E+01 | 1.87E+04 | 6.51E+03 | 2.50E+05 | 5.20E+04 | 1.13E+06 | 4.93E+05 | 6.33E+05 | 2.52E+05 | 1.17E+05 | 4.73E+04 |
| rcCap-Env | 1.00E+01 | 1.00E+01 | 4.43E+03 | 3.06E+02 | 4.10E+04 | 2.65E+03 | 3.70E+05 | 3.00E+04 | 3.30E+05 | 9.64E+04 | 1.67E+05 | 7.02E+04 |
| rcCap-NS1 | 2.67E+01 | 1.15E+01 | 3.20E+04 | 1.01E+04 | 4.33E+05 | 7.02E+04 | 7.67E+05 | 3.79E+05 | 4.67E+04 | 1.53E+04 | 2.13E+04 | 1.02E+04 |

Huh-7 cells, Statistical analysis of mean viral titre relative to wildtype virus control

| DENV2 clone | Days post infection | | | | | |
| --- | --- | --- | --- | --- | --- | --- |
|  | 1 | 2 | 3 | 4 | 5 | 6 |
| rcCap-prM | ** | *** | *** | ** | *** | *** |
| rcCap-Env | *** | *** | *** | n.s. | *** | *** |
| rcCap-NS1 | * | *** | ** | n.s. | n.s. | n.s. |

HepG2 cells, Mean viral titre (PFU/ml)

| DENV2 clone | Days post infection | | | | | | | | | | | |
| --- | --- | --- | --- | --- | --- | --- | --- | --- | --- | --- | --- | --- |
|  | 1 | | 2 | | 3 | | 4 | | 5 | | 6 | |
|  | Mean | SD | Mean | SD | Mean | SD | Mean | SD | Mean | SD | Mean | SD |
| Wildtype | ND | ND | 1.27E+02 | 4.04E+01 | 1.50E+03 | 5.29E+02 | 8.67E+03 | 2.08E+03 | 4.60E+04 | 1.00E+03 | 1.27E+05 | 5.77E+03 |
| rcCap-prM | ND | ND | 1.00E+02 | 1.73E+01 | 1.43E+02 | 3.06E+01 | 1.60E+02 | 3.61E+01 | 1.70E+03 | 1.00E+02 | 2.23E+03 | 1.53E+02 |
| rcCap-Env | ND | ND | 1.33E+01 | 1.53E+01 | 1.67E+01 | 2.08E+01 | 3.00E+01 | 1.00E+01 | 2.33E+02 | 5.77E+01 | 1.33E+02 | 5.77E+01 |
| rcCap-NS1 | ND | ND | 1.07E+02 | 2.52E+01 | 8.00E+02 | 1.00E+02 | 3.87E+03 | 7.23E+02 | 1.93E+04 | 3.06E+03 | 3.57E+04 | 4.51E+03 |

HepG2 cells, Statistical analysis of mean viral titre relative to wildtype virus control

| DENV2 clone | Days post infection | | | | | |
| --- | --- | --- | --- | --- | --- | --- |
|  | 1 | 2 | 3 | 4 | 5 | 6 |
| rcCap-prM | N.A. | n.s. | * | *** | *** | *** |
| rcCap-Env | N.A. | * | *** | *** | *** | *** |
| rcCap-NS1 | N.A. | n.s. | n.s. | * | *** | *** |

C6/36 cells, Mean viral titre (PFU/ml)

| DENV2 clone | Days post infection | | | | | | | | | | | |
| --- | --- | --- | --- | --- | --- | --- | --- | --- | --- | --- | --- | --- |
|  | 1 | | 2 | | 3 | | 4 | | 5 | | 6 | |
|  | Mean | SD | Mean | SD | Mean | SD | Mean | SD | Mean | SD | Mean | SD |
| Wildtype | 2.33E+01 | 1.15E+01 | 4.33E+05 | 5.77E+03 | 9.33E+05 | 2.31E+05 | 7.33E+06 | 1.15E+06 | 2.27E+07 | 4.16E+06 | 4.63E+07 | 1.02E+07 |
| rcCap-prM | ND | ND | 4.57E+02 | 5.69E+01 | 2.33E+03 | 4.51E+02 | 2.33E+04 | 3.06E+03 | 9.33E+04 | 5.77E+03 | 2.23E+05 | 3.79E+04 |
| rcCap-Env | ND | ND | 7.67E+03 | 3.79E+03 | 1.37E+04 | 3.06E+03 | 1.10E+05 | 2.00E+04 | 6.67E+05 | 1.53E+05 | 3.50E+06 | 4.36E+05 |
| rcCap-NS1 | ND | ND | 5.33E+04 | 1.53E+04 | 4.67E+04 | 5.77E+03 | 9.00E+05 | 2.00E+05 | 1.03E+07 | 5.13E+06 | 2.93E+07 | 4.93E+06 |

C6/36 cells, Statistical analysis of mean viral titre relative to wildtype virus control

| DENV2 clone | Days post infection | | | | | |
| --- | --- | --- | --- | --- | --- | --- |
|  | 1 | 2 | 3 | 4 | 5 | 6 |
| rcCap-prM | *** | *** | *** | *** | *** | *** |
| rcCap-Env | *** | *** | *** | *** | *** | *** |
| rcCap-NS1 | *** | *** | *** | *** | * | * |

**Supplementary Table S1.** Statistical analysis of growth kinetics of the recoded viruses relative to wildtype virus in cell culture shown in **Figure 2b**. Statistical analysis of mean viral titre (n=3) of the recoded viruses relative to wildtype for each day post infection was performed using one-way ANOVA, and post-hoc analysis was performed using Tukey HSD. Statistical significance is abbreviated as: n.s, not significant; *, P<0.05; **, P<0.01; ***, P<0.001. ND indicates virus titre was below the limit of detection of 10 PFU/ml. SD: standard deviation.
