# Supplementary material for: Flavivirus genome recoding by codon optimisation confers genetically stable in vivo attenuation in both mice and mosquitoes
Source: PLoS Pathog. 2023 Oct 26;19(10):e1011753. doi: 10.1371/journal.ppat.1011753 (PMC10629665; doi:10.1371/journal.ppat.1011753)
Supplement: S2 Table — Statistical analysis of mean viral titre (n = 3) of the recoded viruses relative to wildtype for each day post infection was performed using one-way ANOVA, and post-hoc analysis was performed using Tukey HSD. Statistical significance is abbreviated as: n.s, not significant; *, P<0.05; **, P<0.01; ***, P<0.001. ND indicates virus titre was below the limit of detection of 10 PFU/ml. SD: standard deviation. (DOCX) [file ppat.1011753.s009.docx]

BHK-21 cells, Mean viral titre (PFU/ml)

| DENV2 clone | Days post infection | | | | | | | |
| --- | --- | --- | --- | --- | --- | --- | --- | --- |
|  | 1 | | 2 | | 3 | | 4 | |
|  | Mean | SD | Mean | SD | Mean | SD | Mean | SD |
| Wildtype | 1.20E+05 | 3.46E+04 | 1.55E+06 | 7.07E+04 | 3.40E+05 | 1.04E+05 | 3.13E+05 | 1.63E+05 |
| WT+rsEnv | 4.67E+04 | 1.15E+04 | 1.00E+07 | 0.00E+00 | 6.00E+06 | 2.00E+06 | 1.00E+06 | 0.00E+00 |
| rcCap-Env | 2.00E+02 | 1.00E+02 | 2.67E+04 | 4.04E+03 | 1.30E+06 | 7.94E+05 | 3.07E+05 | 4.93E+04 |
| rcCap-Env+rsCE | 2.33E+02 | 1.15E+02 | 1.20E+05 | 3.00E+04 | 4.33E+06 | 2.00E+06 | 9.67E+05 | 6.11E+05 |
| rcCap-Env+rsCap | 1.67E+02 | 1.15E+02 | 4.67E+04 | 1.15E+04 | 2.00E+06 | 0.00E+00 | 2.00E+05 | 0.00E+00 |
| rcCap-Env+rsEnv | ND | ND | 1.50E+04 | 3.61E+03 | 2.57E+05 | 5.77E+03 | 2.20E+06 | 1.73E+05 |

BHK-21 cells, Statistical analysis of mean viral titre relative to wildtype virus control

| DENV2 clone | Days post infection | | | |
| --- | --- | --- | --- | --- |
|  | 1 | 2 | 3 | 4 |
| WT+rsEnv | *** | *** | *** | ns |
| rcCap-Env | *** | *** | ns | ns |
| rcCap-Env+rsCE | *** | *** | * | ns |
| rcCap-Env+rsCap | *** | *** | ns | ns |
| rcCap-Env+rsEnv | *** | *** | ns | *** |

Huh-7cells, Mean viral titre (PFU/ml)

| DENV2 clone | Days post infection | | | | | | | |
| --- | --- | --- | --- | --- | --- | --- | --- | --- |
|  | 1 | | 2 | | 3 | | 4 | |
|  | Mean | SD | Mean | SD | Mean | SD | Mean | SD |
| Wildtype | 3.67E+06 | 1.00E+06 | 1.70E+07 | 3.00E+06 | 1.17E+06 | 2.52E+05 | 5.33E+03 | 1.53E+03 |
| WT+rsEnv | 3.00E+05 | 1.00E+05 | 2.33E+06 | 1.00E+06 | 3.33E+05 | 2.31E+05 | 5.33E+04 | 3.21E+04 |
| rcCap-Env | 1.00E+03 | 1.41E+02 | 1.30E+06 | 1.73E+05 | 3.37E+06 | 9.71E+05 | 3.97E+04 | 4.44E+04 |
| rcCap-Env+rsCE | 1.33E+03 | 9.29E+02 | 2.13E+05 | 2.08E+04 | 2.33E+06 | 2.08E+05 | 1.30E+05 | 3.61E+04 |
| rcCap-Env+rsCap | 1.73E+03 | 5.77E+01 | 1.70E+06 | 5.20E+05 | 2.00E+07 | 0.00E+00 | 1.10E+05 | 3.46E+04 |
| rcCap-Env+rsEnv | 1.00E+02 | 0.00E+00 | 1.87E+04 | 5.13E+03 | 1.67E+05 | 6.35E+04 | 1.67E+05 | 8.50E+04 |

Huh-7 cells, Statistical analysis of mean viral titre relative to wildtype virus control

| DENV2 clone | Days post infection | | | |
| --- | --- | --- | --- | --- |
|  | 1 | 2 | 3 | 4 |
| WT+rsEnv | *** | *** | ns | ns |
| rcCap-Env | *** | *** | *** | ns |
| rcCap-Env+rsCE | *** | *** | ns | ns |
| rcCap-Env+rsCap | *** | *** | *** | ns |
| rcCap-Env+rsEnv | *** | *** | ns | * |

**Supplementary Table S2**. Statistical analysis of growth kinetics of the recoded viruses relative to wildtype virus in cell culture shown in **Figure 4c**. Statistical analysis of mean viral titre (n=3) of the recoded viruses relative to wildtype for each day post infection was performed using one-way ANOVA, and post-hoc analysis was performed using Tukey HSD. Statistical significance is abbreviated as: n.s, not significant; *, P<0.05; **, P<0.01; ***, P<0.001. ND indicates virus titre was below the limit of detection of 10 PFU/ml. SD: standard deviation.
