# Supplementary material for: Flavivirus genome recoding by codon optimisation confers genetically stable in vivo attenuation in both mice and mosquitoes
Source: PLoS Pathog. 2023 Oct 26;19(10):e1011753. doi: 10.1371/journal.ppat.1011753 (PMC10629665; doi:10.1371/journal.ppat.1011753)
Supplement: S3 Table — Statistical analysis of mean viral titre (n = 3) of the recoded viruses relative to wildtype for each day post infection was performed using one-way ANOVA, and post-hoc analysis was performed using Tukey HSD. Statistical significance is abbreviated as: n.s, not significant; *, P<0.05; **, P<0.01; ***, P<0.001. ND indicates virus titre was below the limit of detection of 10 PFU/ml. SD: standard deviation. (DOCX) [file ppat.1011753.s010.docx]

BHK-21 cells, Mean viral titre (PFU/ml)

| ZIKV clone | Days post infection | | | | | | | | | |
| --- | --- | --- | --- | --- | --- | --- | --- | --- | --- | --- |
|  | 1 | | 2 | | 3 | | 4 | | 5 | |
|  | Mean | SD | Mean | SD | Mean | SD | Mean | SD | Mean | SD |
| Wildtype | 4.00E+05 | 1.41E+05 | 5.00E+05 | 2.83E+05 | 5.33E+05 | 1.70E+05 | 4.33E+05 | 1.70E+05 | 1.27E+05 | 2.05E+04 |
| rcprM-NS3 | 8.33E+03 | 2.05E+03 | 6.00E+05 | 2.83E+05 | 6.00E+05 | 2.16E+05 | 4.00E+05 | 8.16E+04 | 3.67E+05 | 2.36E+05 |
| rcprM-NS5 | 9.33E+03 | 2.05E+03 | 2.10E+05 | 1.34E+05 | 1.30E+06 | 4.97E+05 | 3.33E+05 | 1.25E+05 | 3.67E+05 | 1.25E+05 |
| rcCap-NS3 | 1.67E+01 | 1.25E+01 | 2.90E+03 | 6.48E+02 | 2.27E+04 | 4.71E+02 | 4.33E+05 | 4.71E+04 | 2.33E+05 | 9.57E+04 |
| rcCap-NS5 | ND | ND | 1.67E+03 | 3.09E+02 | 2.40E+04 | 8.49E+03 | 1.27E+05 | 2.36E+04 | 2.50E+05 | 5.35E+04 |

BHK-21 cells, Statistical analysis of mean viral titre relative to wildtype virus control

| ZIKV clone | Days post infection | | | | |
| --- | --- | --- | --- | --- | --- |
|  | 1 | 2 | 3 | 4 | 5 |
| rcprM-NS3 | n.s | *** | n.s | n.s | n.s |
| rcprM-NS5 | n.s | *** | n.s | * | n.s |
| rcCap-NS3 | n.s | *** | n.s | n.s | n.s |
| rcCap-NS5 | n.s | *** | n.s | n.s | * |

Vero cells, Mean viral titre (PFU/ml)

| ZIKV clone | Days post infection | | | | | | | | | |
| --- | --- | --- | --- | --- | --- | --- | --- | --- | --- | --- |
|  | 1 | | 2 | | 3 | | 4 | | 5 | |
|  | Mean | SD | Mean | SD | Mean | SD | Mean | SD | Mean | SD |
| Wildtype | 4.00E+04 | 2.16E+04 | 1.07E+06 | 9.43E+04 | 7.33E+05 | 2.05E+05 | 5.33E+05 | 3.68E+05 | 1.00E+05 | 8.16E+03 |
| rcprM-NS3 | 9.33E+03 | 2.49E+03 | 2.33E+05 | 1.25E+05 | 7.33E+05 | 2.62E+05 | 6.00E+05 | 2.16E+05 | 1.07E+05 | 2.49E+04 |
| rcprM-NS5 | 6.67E+03 | 9.43E+02 | 5.67E+05 | 2.49E+05 | 1.10E+06 | 1.41E+05 | 3.47E+05 | 1.91E+05 | 8.33E+04 | 2.36E+04 |
| rcCap-NS3 | 6.33E+01 | 1.25E+01 | 3.33E+03 | 1.25E+03 | 1.03E+04 | 1.25E+03 | 2.44E+05 | 3.23E+05 | 8.67E+04 | 3.09E+04 |
| rcCap-NS5 | ND | ND | 6.67E+02 | 4.71E+01 | 4.53E+03 | 1.43E+03 | 1.77E+04 | 2.87E+03 | 3.57E+04 | 1.27E+04 |

Vero cells, Statistical analysis of mean viral titre relative to wildtype virus control

| ZIKV clone | Days post infection | | | | |
| --- | --- | --- | --- | --- | --- |
|  | 1 | 2 | 3 | 4 | 5 |
| rcprM-NS3 | * | * | *** | n.s | n.s |
| rcprM-NS5 | * | * | ** | n.s | n.s |
| rcCap-NS3 | ** | ** | *** | ** | n.s |
| rcCap-NS5 | n.s | ** | *** | ** | n.s |

Huh-7 cells, Mean viral titre (PFU/ml)

| ZIKV clone | Days post infection | | | | | |
| --- | --- | --- | --- | --- | --- | --- |
|  | 1 | | 2 | | 3 | |
|  | Mean | SD | Mean | SD | Mean | SD |
| Wildtype | 1.70E+06 | 9.20E+05 | 6.67E+05 | 9.43E+05 | 1.35E+04 | 9.15E+03 |
| rcprM-NS3 | 6.00E+05 | 8.16E+04 | 2.35E+05 | 3.29E+05 | 3.32E+05 | 2.46E+05 |
| rcprM-NS5 | 9.33E+04 | 9.43E+03 | 1.15E+05 | 1.31E+05 | 1.77E+05 | 1.60E+05 |
| rcCap-NS3 | 1.57E+04 | 1.02E+04 | 1.80E+04 | 8.52E+03 | 1.44E+05 | 1.18E+05 |
| rcCap-NS5 | 2.33E+03 | 1.25E+03 | 4.67E+03 | 1.70E+03 | 1.90E+04 | 7.12E+03 |

Huh-7 cells, Statistical analysis of mean viral titre relative to wildtype virus control

| ZIKV clone | Days post infection | | |
| --- | --- | --- | --- |
|  | 1 | 2 | 3 |
| rcprM-NS3 | n.s | n.s | n.s |
| rcprM-NS5 | ** | n.s | n.s |
| rcCap-NS3 | ** | n.s | n.s |
| rcCap-NS5 | ** | n.s | n.s |

C6/36 cells, Mean viral titre (PFU/ml)

| ZIKV clone | Days post infection | | | | | | | | | | | | | |
| --- | --- | --- | --- | --- | --- | --- | --- | --- | --- | --- | --- | --- | --- | --- |
|  | 1 | | 2 | | 3 | | 4 | | 5 | | 6 | | 7 | |
|  | Mean | SD | Mean | SD | Mean | SD | Mean | SD | Mean | SD | Mean | SD | Mean | SD |
| Wildtype | 5.67E+01 | 2.62E+01 | 5.00E+03 | 2.45E+03 | 1.33E+05 | 2.49E+04 | 7.00E+05 | 1.63E+05 | 1.67E+06 | 9.43E+05 | 8.67E+05 | 3.68E+05 | 6.67E+04 | 2.49E+04 |
| rcprM-NS3 | 4.33E+01 | 1.25E+01 | 1.00E+03 | 8.16E+01 | 1.73E+04 | 8.99E+03 | 9.00E+04 | 1.63E+04 | 2.23E+06 | 1.98E+06 | 7.67E+05 | 1.89E+05 | 6.33E+04 | 3.86E+04 |
| rcprM-NS5 | 6.33E+01 | 4.71E+00 | 9.00E+02 | 2.45E+02 | 9.33E+03 | 9.43E+02 | 1.33E+05 | 4.71E+04 | 7.67E+05 | 1.25E+05 | 8.33E+05 | 2.87E+05 | 3.67E+04 | 2.05E+04 |
| rcCap-NS3 | ND | ND | ND | ND | ND | ND | 1.67E+01 | 9.43E+00 | ND | ND | ND | ND | ND | ND |
| rcCap-NS5 | 6.67E+01 | 3.77E+01 | 6.67E+01 | 9.43E+00 | 5.33E+01 | 4.71E+00 | 6.00E+01 | 2.45E+01 | 1.00E+01 | 9.43E+00 | 2.00E+01 | 8.16E+00 | 1.00E+01 | 4.71E+00 |

C6/36 cells, Statistical analysis of mean viral titre relative to wildtype virus control

| ZIKV clone | Days post infection | | | | | | |
| --- | --- | --- | --- | --- | --- | --- | --- |
|  | 1 | 2 | 3 | 4 | 5 | 6 | 7 |
| rcprM-NS3 | n.s | ** | *** | *** | n.s | n.s | n.s |
| rcprM-NS5 | n.s | ** | *** | *** | n.s | n.s | n.s |
| rcCap-NS3 | n.s | ** | *** | *** | n.s | ** | * |
| rcCap-NS5 | n.s | ** | *** | *** | n.s | ** | * |

**Supplementary Table S3**. Statistical analysis of growth kinetics of the recoded viruses relative to wildtype virus in cell culture shown in **Figure 5b**. Statistical analysis of mean viral titre (n=3) of the recoded viruses relative to wildtype for each day post infection was performed using one-way ANOVA, and post-hoc analysis was performed using Tukey HSD. Statistical significance is abbreviated as: n.s, not significant; *, P<0.05; **, P<0.01; ***, P<0.001. ND indicates virus titre was below the limit of detection of 10 PFU/ml. SD: standard deviation.
